# Structural Dynamics Predominantly Determine the Adaptability of Proteins to Amino Acid Deletions

**DOI:** 10.3390/ijms24098450

**Published:** 2023-05-08

**Authors:** Anupam Banerjee, Ivet Bahar

**Affiliations:** 1 Laufer Center for Physical and Quantitative Biology, Stony Brook University, Stony Brook, NY 11794, USA; 2 Department of Biochemistry and Cell Biology, Stony Brook University, Stony Brook, NY 11794, USA

**Keywords:** adaptability, deletion mutations, folding stability, positive-unlabeled learning classifiers, structural dynamics, elastic network models

## Abstract

The insertion or deletion (indel) of amino acids has a variety of effects on protein function, ranging from disease-forming changes to gaining new functions. Despite their importance, indels have not been systematically characterized towards protein engineering or modification goals. In the present work, we focus on deletions composed of multiple contiguous amino acids (mAA-dels) and their effects on the protein (mutant) folding ability. Our analysis reveals that the mutant retains the native fold when the mAA-del obeys well-defined structural dynamics properties: localization in intrinsically flexible regions, showing low resistance to mechanical stress, and separation from allosteric signaling paths. Motivated by the possibility of distinguishing the features that underlie the adaptability of proteins to mAA-dels, and by the rapid evaluation of these features using elastic network models, we developed a positive-unlabeled learning-based classifier that can be adopted for protein design purposes. Trained on a consolidated set of features, including those reflecting the intrinsic dynamics of the regions where the mAA-dels occur, the new classifier yields a high recall of 84.3% for identifying mAA-dels that are stably tolerated by the protein. The comparative examination of the relative contribution of different features to the prediction reveals the dominant role of structural dynamics in enabling the adaptation of the mutant to mAA-del without disrupting the native fold.

## 1. Introduction

The insertion/deletion (indel) of nucleotides is an important source of genetic variation. Those occurring at coding regions via triplets of nucleotides, i.e., non-frame-shifting indels, result in amino acid indels (AA-indels). AA-indels constitute a mechanism of protein evolution alongside point mutations [1,2,3,4]. While point substitutions alter the side chains of residues, indels alter the protein backbone. Such modifications can have a wide range of effects, from disease conditions [5] to benefits in terms of structure or function [6,7]. While most point mutations are neutral, indels and especially those involving multiple amino acids (AAs), called mAA-indels, may introduce significant leaps in the fitness landscape [1] and drive the evolutionary adaptation to new functions [8,9,10]. AA-indels contribute to approximately one-fourth of disease-causing mutations in humans [5,11] and are responsible for several Mendelian disorders [12] and different types of cancers [13]. They are also responsible for the functional divergence between homologous protein structures [14]. For example, a polybasic insert near the S1/S2 cleavage site functionally distinguishes the SARS-CoV-2 spike protein from its orthologs in the SARS subfamily of betacoronaviruses [10].

There have been many experimental studies on the effects of pre-defined AA-indels on catalytic specificities [15,16] or on the binding affinities of engineered antibodies [17,18]. Despite the recognized importance of accurately assessing the effects of AA-indels on structure and function, the use of machine learning (ML) tools for accomplishing this goal has been limited, and backbone modifications for protein engineering purposes remain relatively less explored [19].

AA-indels frequently occur at loops/coils, as these regions more readily sustain variations than buried or structured regions. Such observations directed attention to investigating the thermodynamic stability or native state entropy of AA-indel mutants [20,21], the role of compensatory mutations in supporting structural stability [1,22], the effect of point mutations and backbone modifications on the evolution of structure [23], and the pathogenicity of indels [5,24,25,26]. However, a computational framework to differentiate between destabilizing and neutral AA-indels remains to be established.

The primary impediment to constructing ML-based predictors has been the sparsity of learning data. However, recent studies have succeeded in compiling datasets and developing classifiers that estimate the foldability of proteins containing single-point [27] and multiple (contiguous) deletions [28]. In these studies, hundreds of AA-indels of various sizes (up to *n_AA_* = 23 amino acids) have been identified that retain the wild-type (wt) fold in the absence of compensatory mutations.

In the present study, we build on this recent progress to establish the molecular basis for the ability of proteins to accommodate or resolve mAA-dels with minor, if any, changes in wt structure. As will be shown below, the intrinsic dynamics of the protein, not considered in previous studies, emerge as a major determinant of adaptability to mAA-dels and help discriminate between sustainable mAA-dels and those that would compromise the native fold.

Intrinsic dynamic refers to the collective modes of molecular motions evolutionarily optimized and uniquely encoded by the native fold, which usually enable protein–protein interactions, allosteric signaling, or other activities [29,30,31]. The structure-based modeling of protein dynamics has been successfully incorporated into previous ML-based algorithms for inferring the mechanisms of protein function [32]. The efficient evaluation of intrinsic dynamics using elastic network models (ENMs) [33,34] also proved useful in the genome-scale characterization of biomolecular dynamics [34], the ensemble analysis of protein families [35], or the ML-based prediction of pathogenicity for single-amino-acid variants [36,37,38]. The latter highlighted the role of intrinsic dynamics in eliciting or avoiding a pathogenic response.

The present study shows that the inclusion of dynamics-based features in a new positive-unlabeled (PU)-learning classifier yields a high recall rate of 84.3%. Notably, among various sequences, structures, and dynamics features considered in the algorithm, the dynamics features predicted by ENMs contribute by 72.3% to classification; and among ENM-based features, the collective motions of the native fold at the two ends of the spectrum (i.e., the lowest and the highest frequency modes) are distinguished as major determinants of the effect of mAA-dels. Furthermore, the involvement in allosteric communication is noted as another important feature that precludes the adaptation to the mAA-del. Overall, this analysis points to the intrinsic dynamics of the overall wt protein, and not that of the local structure or the chemical features at the AA-indel alone, as the major determinant of adaptability to mAA-dels.

## 2. Results

### 2.1. Dataset

We adopted a dataset [28] previously compiled for assessing the stability of deletion mutants using a PU classifier, *Profound.* As described in the methods, the dataset contains data for 153 proteins deposited in the Protein Data Bank (PDB) as both wt and mutants containing mAA-dels of length 2 ≤ *n_AA_* ≤ 23 (called the subset of positive mAA-dels), as well as a curated set of conformers (7649 of them) derived from existing PDB structures where indels were randomly introduced (called the subset of unlabeled mAA-dels). In the former subset, the mutant structure (deposited in the PDB) exhibits at least 70% fold similarity to that of the wt protein as computed by TM-align [39]. Unlabeled mAA-dels, on the other hand, refer to the cases where the effects of indels on the fold are unknown.

### 2.2. Features Describing the Intrinsic Dynamics of the Protein

**Figure 1:**
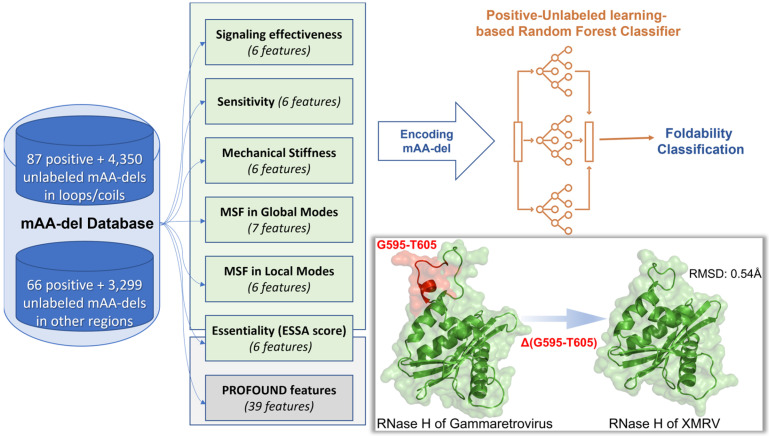
Schematic of the proposed method illustrating the database and features used to construct the PU-learning classifiers. The inset shows an example of a positive mAA-del in which the protein (RNase H of Gammaretrovirus, PDB ID: 4E89, Chain A, containing the Gly595–Thr605 stretch) and its homolog with the mAA-del at that particular stretch (RNase H of XMRV, PDB ID: 3V1Q, Chain A) exist both in nature. An RMSD of 0.54 Å between the two structures shows that the deletion of the Gly595–Thr605 stretch does not affect the folded conformation.

We considered six types of features based on intrinsic dynamics, all evaluated for the wt protein using the *ProDy* interface [40,41] (Figure 1): (i) the mean-square fluctuations (MSFs) of residues along the softest collective motions (*global modes*) predicted by the Gaussian network model (GNM) [42]; softest modes here refer to the 2% of the *N*-1 GNM modes lying at the lowest frequency end of the mode spectrum accessible to a protein of *N* residues; these are robustly defined by the overall architecture of the protein; (ii) MSFs along the same number of highest frequency GNM modes (*local modes*); (iii) *sensitivity* of amino acids to perturbations; (iv) *effectiveness* to transduce signals within the structure—the latter two provide a measure of the role of deleted residues in sensing or transmitting allosteric signals prior to deletion, and they are deduced from perturbation response scanning (PRS) analysis [43] as previously described [44,45]—(v) *mechanical stiffness* of mAA-del as measured by the effective resistance to uniaxial tension [46]; and (vi) *essentiality* of mAA-del prior to deletion, as predicted by the essential site scanning analysis (*ESSA*) [47]. A higher *ESSA* score means a higher capacity to alter the global dynamics if bound to a ligand.

For each of the six features, we computed six values: the minimum and maximum values among the mAA-del residues, and the mean, in addition to the corresponding minimum and maximum *Z-scores* and the mean *Z-score*, <*Z-score*>. Additionally, using the *global mode* shape, we estimated whether the mAA-del is co-localized with a hinge site. This led to 37 features for each mutant, which were positive or unlabeled.

### 2.3. Dynamics-Based Features Discriminate between Positive and Unlabeled mAA-dels

**Figure 2:**
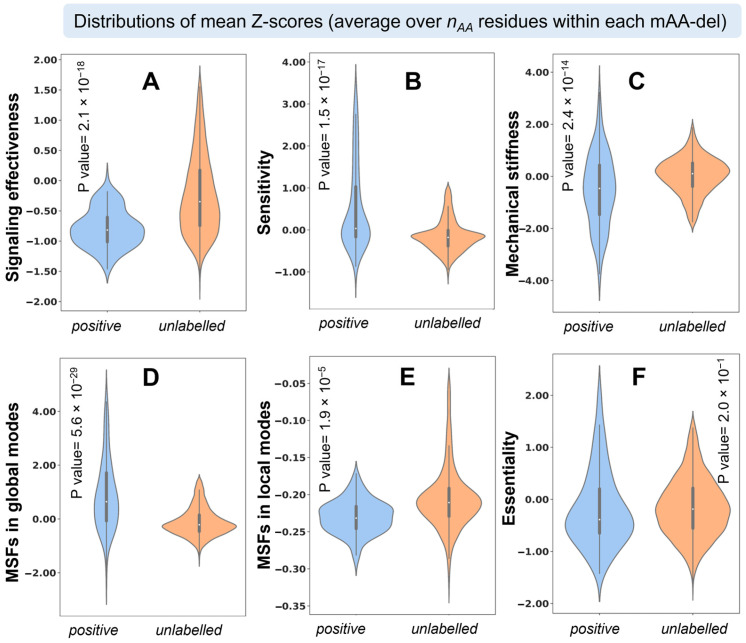
Dynamics-based features are differentially distributed in the positive and unlabeled mAA-del subsets. Violin plots show the distribution of <*Z-scores*> (averaged over *n_AA_* residues for each mAA-del) for (**A**) *effectiveness*, (**B**) *sensitivity*, (**C**) *mechanical stiffness*, (**D**) *MSFs* in *global modes*, (**E**) *MSFs* in *local modes*, and (**F**) *essentiality* (*ESSA score*) for the subsets of positive (blue) and unlabeled (orange) mAA-dels.

Figure 2 displays the <*Z-scores*> computed for the six dynamics-based properties listed above for the subsets of positive and unlabeled deletions. More detailed distributions broken down by loop and non-loop regions are presented in the Supplementary Figure S1. The counterparts of Figure S1 for the maximum and minimum *Z-scores* are presented in the respective Supplementary Figures S2 and S3. The distributions for the unlabeled mAA-dels act as a control for the statistical significance of the positive mAA-del features. We found that all dynamics-based features considered here, except *essentiality*, significantly differ between the positive and unlabeled/control mAA-dels. This is evidenced by the *p*-values reported in Figure 2, as well as the results from Student’s *t*-test, Welch two-sample *t*-test, and two-sample *Z*-test (Tables S1–S4). *Essentiality* also contributes to the classification of mAA-dels, as will be shown below; however, its contribution is relatively smaller. The violin plots in Figures S1–S3 further show that the loop and non-loop regions exhibit distinctive dynamics.

### 2.4. Enhanced Mobilities in Global Modes Underlie the Adaptation of wt Structure to mAA-dels

**Figure 3:**
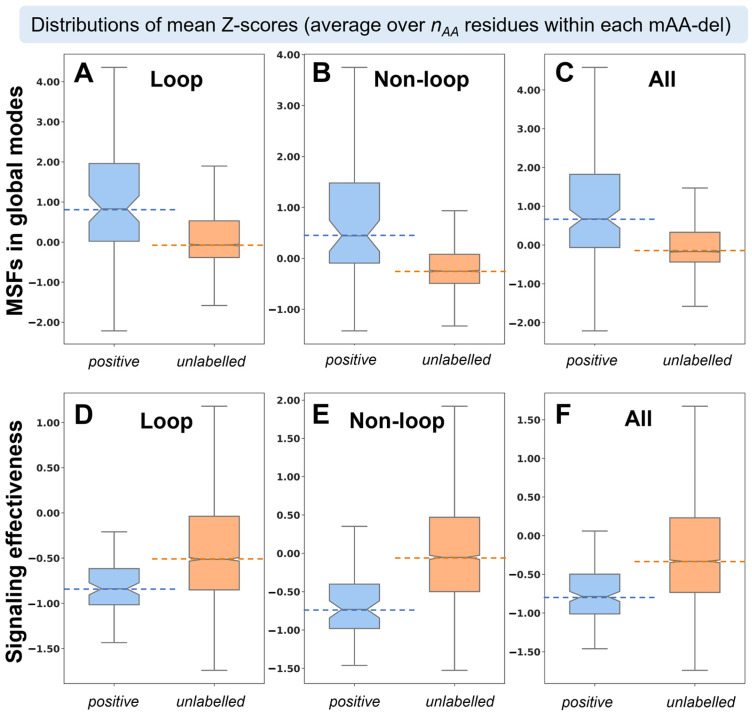
*MSFs* in *global modes* and signaling *effectiveness* distinguish the subsets of positive and unlabeled mAA-dels. Notched box plots show the distribution of <*Z-scores*> corresponding to *MSFs* in *global modes* for mAA-dels in (**A**) loop, (**B**) non-loop, and (**C**) all regions for positive (blue) and unlabeled (orange) mAA-dels. The distributions for the *effectiveness* of mAA-del residues in (**D**) loop, (**E**) non-loop, and (**F**) all regions further demonstrate the differences between the positive and unlabeled subsets.

The above analysis reveals the significance of the fluctuations in global modes in distinguishing between positive and unlabeled mAA-dels. Figure 3A–C display the notched box plots for the MSFs of the *n_AA_* mAA-del residues in the global modes (prior to deletion) averaged over the *n_AA_* residues. Residues belonging to positive mAA-dels exhibit a broad range of fluctuations, and generally exhibit higher fluctuations in global modes compared to those belonging to the unlabeled mAA-dels. This means that deletions at regions that enjoy relatively large movements in the global modes are more readily accommodated by the protein structure. The same effect is apparent in both loop and non-loop regions. The respective median values for positive mAA-dels are 0.83 and 0.44; and those for the unlabeled mAA-dels are −0.07 and −0.25. Furthermore, the fluctuations in unlabeled deletions are narrowly distributed, which further indicates the higher constraints experienced by these deletions compared to those of the positive mAA-dels. The Welch two-sample *t*-test and Student’s *t*-test and *Z*-test results (Table S2) further corroborate the significance of conformational mobility in global modes as a determinant of the adaptability of the 3D structure to the deletion.

### 2.5. Deletion of Effectors of Allosteric Communication Impairs the Adaptation to mAA-dels

This is evidenced by the higher occurrence of signaling *effectiveness* in unlabeled mAA-dels than positive mAA-dels (Figure 3D–F). Figure 3D shows that the signaling *effectiveness* (<*Z-score*> median of −0.84) of positive mAA-dels is much lower than that (−0.51) of the unlabeled mAA-dels for the loop regions, and the differences are further pronounced (−0.71 vs. −0.05) in the non-loop regions. We observe a similar trend for the consolidated set (Figure 3F) where the median values for positive and unlabeled mAA-dels are −0.79 and −0.33, respectively. This means that the residues participating in positive mAA-dels exhibit a lower tendency to transmit/propagate allosteric signals across the structure than other residues, which may explain their accommodation without necessitating an alteration in 3D fold. The Welch two-sample *t*-test (and Student’s *t*-test and *Z*-test) data (Table S2) further support this result. Additionally, we observe that the third quartile of the positive instances in the loop, non-loop, and ‘all’ regions in Figure 3D–F are well below the zero value. All these data robustly establish that mAA-dels that are sustained without alteration in the native structure are minimally involved, if any, in signal transmission, and hence these non-influential (in allostery) residues can be resolved without the need for introducing changes in the native fold.

### 2.6. Relatively Lower Mechanical Stiffness at mAA-del Site Assists in Adaptation

*Mechanical stiffness* is a metric that helps us quantify the effective resistance of residue pairs to uniaxial tension [46]. Residues belonging to positive mAA-dels are observed to be broadly distributed and show overall lower *mechanical stiffnesses* compared to those belonging to unlabeled deletions (see Figure 2C). This is consistent with the fact that a higher *mechanical stiffness* would confer a stronger resistance to accommodate the deletion. The median of the *mechanical stiffness* <*Z-score*> for the positive mAA-dels in loops (−0.65) is significantly weaker than that of the unlabeled mAA-dels (0.04), and similar trends (−0.46 vs. 0.11 and −0.49 vs. 0.08) have been observed in the non-loop and ‘all’ regions, respectively. Similarly, the Welch two-sample *t*-test and *Z*-test (Table S2) indicate significant (*p* < 0.001) differences. These data confirm that lower resistance to uniaxial tension (or lower stiffness) is a discriminating feature of positive mAA-dels.

### 2.7. Deletion of Residues Involved in High-Frequency (Local) Motions Disrupts the Native Fold

High-frequency modes usually induce highly localized fluctuations in the most tightly packed (e.g., core) regions of proteins. These regions, also referred as kinetically hot sites (due to the localization of high vibrational energy), have been proposed to serve as folding nuclei [48,49], and would be expected to resist mutations, including deletions. The peaks in the residue-profile of *MSFs* in *local modes* thus help us identify such highly conserved and tightly packed residues. The median values for <*Z-score*> associated with *MSFs* in *local modes* were −0.23 (loop), −0.22 (non-loop), and −0.23 (all) for positive mAA-dels, which are all lower than their counterparts for unlabeled mAA-dels (Figure S1E). Therefore, positive mAA-dels seldom contain kinetically hot residues. The Welch two-sample *t*-test (Table S2) also yields a significant difference between the two subsets regardless of their location (loop or non-loop). The deletions of such segments involved in high-frequency fluctuations are therefore not tolerated due to their frequent participation in core interactions that stabilize the structure.

### 2.8. Classifiers Exclusively Trained on Intrinsic Dynamics Yield High Recall and Low Fall-Out Rates

To quantify the predictive ability of classifiers that distinguish between positive mAA-dels and others, we performed three sets of computations, using (a) the dynamics-based features computed using *ProDy*; (b) the sequence and structure-dependent features adopted in *Profound*; and (c) the combination of the two sets. In all cases, we trained three classifiers specific to loop, non-loop, and all regions. The stratified 10-fold (and 5-fold) cross-validation of the PU-learning classifiers ensured the proportionate representation of mAA-dels from the positive and unlabeled class labels to train and test the classifiers. In each cross-validation, we evaluated the recall and fall-out rates as metrics to evaluate the performance of the classifier and the probabilistic occurrence of positive mAA-dels within the unlabeled dataset. The recall rate informs us of the percentage of correctly identified positive mAA-del instances; whilst the fall-out rate measures the percentage of unlabeled mAA-dels that are predicted to be positive. In the absence of information regarding the true positive and true negative instances among the unlabeled mAA-dels, the fall-out rate provides an estimate of the expected fraction of positive mAA-dels among randomly selected mAA-dels.

**Figure 4:**
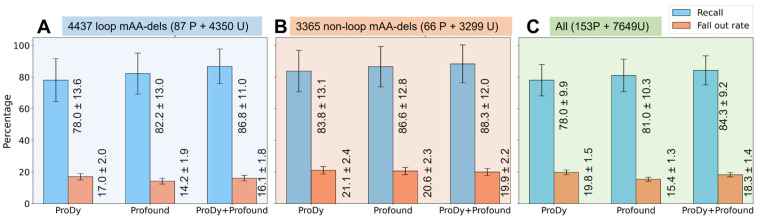
High performance of PU-learning classifiers for predicting positive mAA-dels. Results are presented from stratified 10-fold cross-validations for loops, non-loop regions, and all regions in the respective panels (**A**–**C**). The recall (blue bars) and fall-out rates (orange bars) are displayed for the classifiers trained on dynamics-only (*ProDy*) features (left bar), *Profound*-only features (middle bar) and consolidated (*ProDy* + *Profound*) features (right bar) for mAA-dels in loops (**A**), non-loop regions (**B**), and all regions (**C**).

The leftmost pair of bars in each of the three panels A–C of Figure 4 show that classifiers exclusively trained on dynamics features (labeled as *ProDy*) report a recall of 78.0% and a fall-out rate of 17.0% (during stratified 10-fold cross-validation) for mAA-dels in loops (panel A); the respective percentages are 83.8% and 21.1% in non-loop mAA-dels (panel B); and 78.0% and 19.8% in the combined dataset (panel C). The standard deviations are lower in the combined set compared to those in panels A and B. The counterparts of these bars for stratified 5-fold cross-validations confirm the same trend (Table S3). These data reveal the ability of classifiers exclusively trained on dynamics-based features to distinguish between positive and unlabeled mAA-dels. Their performance is only slightly below that of the state-of-the-art classifier *Profound* (middle pairs of bars in panels A–C of Figure 4) despite the use of a large and diverse set of features in the latter, including sequence-, structure-, environment-dependent, and physicochemical features.

### 2.9. Inclusion of Dynamics Features Improves the Ability of State-of-the-Art Random Forest (RF) Classifier to Predict the Effect of mAA-dels on Fold Stability

Next, we examined whether the PU-learning classifiers constructed with the consolidated feature set (referred to as *ProDy + Profound*) outperformed those based on either set of features. To have a common set of consolidated features, we omitted two loop-specific features used in the *Profound* classifier specific to loop mAA-dels. We find that, on stratified 10-fold cross-validation, the PU-learning classifiers trained on the consolidated feature set report a recall of 86.8% for mAA-dels in loops, and 88.3% in non-loop regions; the respective fall-out rates are 16.1% and 19.9 (rightmost bars in Figure 4A,B); and in the case of all mAA-dels (Figure 4C), the two percentages and their standard deviations are 84.3 ± 9.2 and 18.3 ± 1.4. In all three cases, the consolidated (*ProDy + Profound*) classifiers outperform the individual classifiers, indicating that the merged set of features in the RF enables a more accurate identification of positive mAA-dels. The recall rate exceeds those of the separate classifiers by 2–8 percentage points (depending on the subsets of mAA-dels).

The fall-out rate provides important information—that of success (in maintaining the native fold) by randomly occurring mAA-dels during evolution. A value of 15–20% is computed regardless of the classifier, suggesting that one out of 5–6 randomly selected mAA-dels is likely to be accommodated by the protein. In other words, the observed positive mAA-dels in the PDB presumably represent 1/5–1/6 of all possible mAA-dels, the large majority of which have not survived, presumably giving rise to fold destabilization.

Overall, the inclusion of dynamics-based features in the combined dataset helps us construct classifiers with improved predictive power (regarding the effect of mAA-dels on folding stability) and provide estimates of the fraction of mAA-dels (80–85%) that are not evolutionarily sustained. We next examine which features dominate the outcomes when all are used to train the RF classifier.

### 2.10. Dynamics-Based Features Predominantly Determine the Change in Folding Stability Caused by mAA-dels

The use of decision tree-based RFs enables us to quantify the contribution of individual features to classification. We present in Table S4 the contributions to both 5-fold and 10-fold cross-validations for PU-learning classifiers constructed for mAA-dels in loops, non-loop, and all regions, based on *ProDy* and on *ProDy + Profound* features.

**Figure 5:**
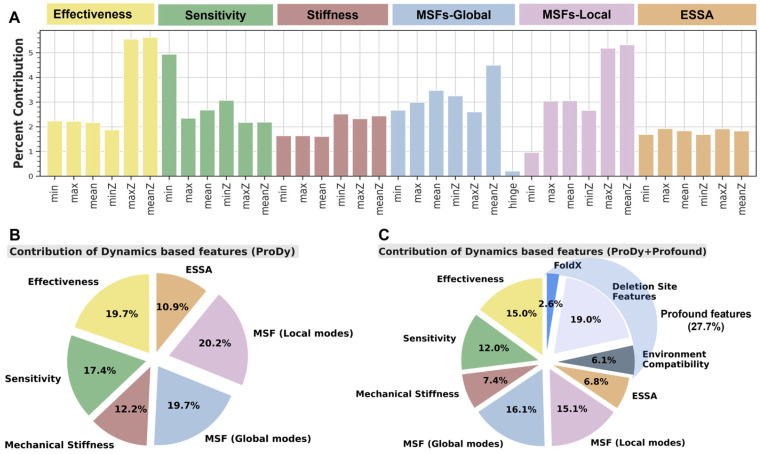
Dynamics-based features are major determinants of the ability of mAA-del-containing mutants to retain the native fold. (**A**) The percent contribution of individual features to stability classification as assessed by the stratified 10-fold cross-validation of the PU-learning classifier exclusively trained on dynamics-based features. The attributes are color-coded based on the feature. (**B**,**C**) Agglomerated contribution of different features when the classifier is trained only on dynamics-based features (**B**), or on the merged feature set (**C**).

First, we examine the relative contributions of dynamics-based features. Figure 5A,B display the corresponding results from 10-fold cross-validation. Panel A displays the contributions of features broken down by their various values/scores, as listed along the abscissa, and the pie chart in panel B displays the agglomerated contribution of each feature. The MSFs of mAA-del residues in the global (19.7%) and local (20.2%) modes of the wt protein contribute almost 40% to foldability classification, followed by *effectiveness* to propagate signals/interactions (19.7%) and *sensitivity* to signals/interactions (17.4%). Even *ESSA* scores contribute about 11%, despite their relatively high *p*-values (see Figure 2). These dominant features are invariably distinguished in either the *ProDy* only or *ProDy* + *Profound* classifiers (Tables S4 and S5). The distributions of the minimum *sensitivity*, *MSF* (*local modes*) <*Z-score*>, *effectiveness* <*Z-score*>, and the *MSF* (*global modes*) <*Z-score*> for positive and unlabeled mAA-dels in the merged dataset are presented in Figure S4A–D, respectively.

Next, we turn our attention to the contributions of all features to the classifiers generated for the consolidated feature set (Figure 5C). Strikingly, dynamics-based (*ProDy*) features contribute 72.3%. These are by far the largest contributors to the classification process, even in the presence of *Profound* features. This further calls for attention to the significance of intrinsic dynamics attributes as the major determinant of the adaptability of protein folds to mAA-dels. Notably, in Figure 5C, the relative contributions of the six dynamics-based features exhibit similar trends to those observed in panel B, indicating the robustness of the relative importance of these features. As regards *Profound* features, a group designated as ‘deletion site features’ makes the largest (19%) contribution. The group includes information on hydrogen bonds, salt bridges, solvent accessibility, dihedral angles, end-to-end distance, and amino acid frequencies relative to natural occurrences, specific to the residues lying within the mAA-del.

## 3. Discussion

Multiple ML methods have been proposed in the last decade for predicting the pathogenicity associated with indels, including the SIFT Indel [26], DDIG-in [50,51], VEST-Indel [52], and MutPred-LOF/-Indel [25,53] methods trained using the HGMD [11]. These methods are based on gene and/or sequence information (structural features, when used, are predicted from sequence). On the other hand, structural data are essential to make inferences on biophysical effects. Indel PDB [54] and IndelFR [55] are structural databases of indels identified from the sequence alignments of highly similar proteins found in the PDB, but the aligned sequences are not necessarily sequentially identical and may contain compensating mutations [1], which may complicate the interpretation of the response to AA-dels. Here, we considered pairs of protein structures whose sequences are identical, except for the mAA-del, which enabled us to sort out which properties of the wt protein predominantly underlie the adaptation to mAA-dels.

Our study highlights the importance of the protein intrinsic dynamics in defining the adaptability to mAA-dels. Strikingly, classifiers *exclusively* trained on dynamic properties (using *ProDy*) achieve a recall rate of 78%. When combined with other features (39 of which are adopted in *Profound*, including the sequence, structure, and environmental features), the improvement is relatively modest (to 84%). However, the dynamics-based features make a major contribution (72.3%) to the prediction. A dichotomy is the success (81%) of *Profound*, even though its features contribute only 27.7% to the predictor when used together with *ProDy* features. Given that intrinsic dynamics are themselves dependent on structure, which is also encoded by sequence, it is conceivable that *ProDy* supersedes many features otherwise included in *Profound*, but not all of them. However, the significantly higher weights assigned to *ProDy* features when all features are included in training the RF predictor point to the relatively stronger power of intrinsic dynamics for distinguishing the mAA-dels that can be tolerated vs. others. In this context, it is important to note that the proposed classifiers might fall short of identifying the impact of mAA-dels in intrinsically disordered proteins (IDPs) as elastic network models are unable to accurately describe and quantify the intrinsic dynamics of IDPs.

Previous studies have demonstrated that intrinsic dynamics provide the mechanisms for accomplishing biological function, adapting to protein–protein interactions, or defining the response to single amino acid variants. The present study further shows that intrinsic dynamics underlie the adaptability to short deletions (up to 23 amino acids included herein). Not only do dynamics-based features differentiate between positive and unlabeled mAA-dels, but they also play a dominant role in predicting positive mAA-dels (see Figure 5C). Note that the dynamics-based features considered herein are agnostic to sequence and are purely based on the 3D structure of the wt protein modeled as an elastic network, without any knowledge of the specific interactions and or energetics. They are purely defined by the topology of inter-residue contacts in the native structure. As such, they reflect the preferences driven by conformational entropy in the native state. The computed MSFs and other ENM-dependent properties represent unique solutions that comply with the entropy maximization principle for the collective fluctuations of all residues near native state conditions [56].

Finally, the present study was possible despite the sparsity of data on mAA-del containing proteins/mutants because of the use of a PU-learning-based classifier. PU-learning-based classifiers have recently been used in several biological applications, including the prediction of drug–drug interactions [57,58] and the identification of RNA disease associations [59]. The present study demonstrates their utility in assessing the effect of deletions on folding stability. The database was constructed by extracting from the PDB pairs of proteins or chains/subunits (in the case of multimeric structures) that shared the same sequence except for the mAA-del in one of them. However, it is important to note that, in certain cases, the extracted subunit might not fold in the absence of the other subunits of the multimeric protein or complex; and consequently, the structural and dynamic features evaluated for such cases might not be in compliance with the adaptability to deletions. To assess the extent to which such effects might have affected the results, we thoroughly examined whether the monomers/subunits used in our dataset were sufficiently stable to exist as monomers. Our extensive survey, compiled in Supplementary Table S6, showed evidence of existence as monomers for 86% of our positive mAA-dels. Furthermore, on the flip side, the inclusion of the effect of the interacting units in the environment might make it difficult to discern the effects of mAA-dels themselves on the folding properties of individual proteins/subunits. Furthermore, one major constraint in studying mAA-dels has been the sparsity of data. The inclusion of a small fraction of subunits that may not be stable in isolation is a compromise to increase the population of positive mAA-dels. With the increase in structural data on deletion mutants and the possibility of using a dataset containing the wt and mutant in the same multimerization or complexation state, we anticipate the performance of the PU-learning classifier to be higher.

Another interesting piece of information provided by this study was the fall-out rates of 15–20%, which provides us with an estimate of the naturally occurring fraction of positive mAA-dels. This type of information cannot be observed since proteins subject to negative mAA-dels are not evolutionarily sustained, even if such deletions occur 4–5 times more frequently than the positive mAA-dels.

Finally, we recognize that the introduction of AI-driven structure prediction methods such as AlphaFold2 [60] and RoseTTAFold [61] has been revolutionary. However, as recently pointed out by Buel and Walters, AlphaFold2 is insensitive to structure-disrupting mutations in an input sequence as there are no databases that provide structural information on mutations, and hence, the predictions are largely based on wt or homologous sequences [62]. In silico predictors of the effects of genome variants provide new therapeutic opportunities in personalized medicine [63]. These studies focus on different types of mutations and their various effects from folding stability to disease-causing properties. Here, we focused on deletions and developed a framework for predicting their effects on native fold stability. Such predictors, which will only improve with increasing data, can help open new avenues for protein engineering and molecular therapeutics.

## 4. Materials and Methods

### 4.1. Dataset

We adopted the dataset [28] previously compiled to prepare the PU classifiers in *Profound*, accessible at https://cse.iitkgp.ac.in/~pralay/resources/PROFOUND/, accessed on 5 May 2023. The dataset contains 153 positive and 7649 unlabeled mAA-dels. Unlabeled mAA-dels obey the same distributions (for *n_AA_*, the fraction of loop residues being deleted *n_AA_*/*n_loop_*, where *n_loop_* is the number of residues in the loop/coil, and the location of a deleted segment with respect to *N*- or *C*-termini) as the proteins in the subset of positive mAA-dels. Deletions take place at any position along the protein sequence except for the *N*- and *C*-termini. The positive mAA-dels belonged to proteins from 42 different species and 5 different SCOP classes. We also noted that 132 out of 153 (~86%) positive mAA-dels belong to proteins that have been observed as monomeric units (see Table S6). The wt and mutant proteins for the remaining 21 mAA-dels both belong to identical multimeric assemblies. We further examined the subsets of mAA-dels located in loops (87 positive and 4350 unlabeled) and other regions (66 positive and 3299 unlabeled).

### 4.2. Intrinsic Dynamics-Based Attributes

*MSFs of residues in the global and local modes.* We used the Gaussian Network Model (GNM) [42,64] to compute the MSFs driven by the global modes, using 2% of the N-1 GNM modes at the lowest frequency end of the mode spectrum (*MSF global modes*). Secondly, the MSFs driven by the same number of highest frequency GNM modes (*MSF local modes*) were computed. In the GNM, the cross-correlation between the fluctuations $\Delta R_{i}$ and $\Delta R_{j}$ in the position vectors $R_{i}$ and $R_{j}$ of C^α^-atoms i and j scales with the *ij*th element of the pseudoinverse of the Kirchhoff connectivity matrix **Γ**, as-

(1) $$\langle \Delta R_{i}\;\cdot \;\Delta R_{j}\rangle =\left(k_{B}T/\gamma \right){\left[\Gamma^{-1}\right]}_{ij}$$

where $k_{B}$ is the Boltzmann constant, T is the absolute temperature, and γ is the uniform force constant between all nodes of the GNM representing the 3D structure [65]. The ijth element of Γ is −1 if the distance between the C^α^-atoms i and j is less than a cutoff distance (usually 10 Å) of direct interaction; and the *i*th diagonal element is the coordination number of that residue (or the degree of the network node at the *i*th C^α^-atom). The MSFs of residue *i*, $\langle {\left(\Delta R_{i}\right)}^{2}\rangle ,$ are obtained by using *i = j* in Equation (1), and may be broken down into the contribution of the normal modes *k* as

(2) $$\langle {\left(\Delta R_{i}\right)}^{2}\rangle =\left(k_{B}T/\gamma \right){\left[\Gamma^{-1}\right]}_{ii\;}=\left(k_{B}T/\gamma \right){\left[\sum_{k=1}^{N-1}\lambda_{k}^{-1}u_{k}u_{k}^{T}\right]}_{ii}$$

where $u_{k}$ and $\lambda_{k}$ designate the *k*^th^ eigenvector and eigenvalue of Γ. The modes are usually organized in ascending order, such that mode 1 refers to the lowest frequency (most global) motion, and mode *N*-1 describes the highest frequency (most local) motion. In a protein of *N* = 200 residues, for example, modes 1 ≤ *k* ≤ 4 are included in eq 2 to calculate the *MSFs* in *global modes*, and modes 196 ≤ *k* ≤ 199 define the *MSFs* in *local modes*.

*Ability to serve as sensors and effectors of allosteric communication*. We used the PRS module [44,66] of the *ProDy* API to compute the *sensitivity* and *effectiveness* of residues with regard to the sensing and transmitting signals which typically propagate through coupled fluctuations in residue positions. PRS uses the linear response theory [67] to sequentially apply directed forces on each residue and compute the resulting change in position of all residues. In principle, for a network of elastic springs, the force–displacement relation is given by **F** = **H** Δ**R**, where **F** is a 3*N*-dimensional force vector, **H** is the Hessian matrix corresponding to the anisotropic network model (ANM), and Δ**R** is the *3N*-dimensional fluctuation $(\Delta R_{1}$ $\Delta R_{2}$ … $\Delta R_{N}$)^T^; conversely, by pre-multiplying both sides by **H**^−1^, the response Δ**R** to **F** becomes

Δ**R** = **H**^−1^
**F** (3)

In the PRS, **F** is the perturbation and Δ**R** is the response. The response to a perturbation at residue *i* may be expressed as $\Delta R^{\left(i\right)}={\left(\Delta r_{1x}^{\left(i\right)}\;\Delta r_{1y}^{\left(i\right)}\;\Delta r_{1z}^{\left(i\right)}\ldots \;\ldots \;\Delta r_{Nz}^{\left(i\right)}\right)}^{T}$. The response (*sensitivity*) of the residue j to the perturbation of residue *i* (effector) is organized in a *N × N* PRS matrix, **S_PRS_**, the *i*th row of which describes the *effectiveness* of the *i*th residue in transmitting signals and the *j*th column corresponds to the *sensitivity* of a residue *j* to signals from other residues. The response to unit perturbation at each site is obtained by dividing each row of **S_PRS_** by its diagonal element. The average of row *i* elements in this normalized matrix represents the *effectiveness* of residue *i*, and the average of column *i* represents the *sensitivity* of residue *i* [44].

*Mechanical stiffness* defines the effective resistance of residue pairs to uniaxial tension. The *mechanical stiffness* was computed using the *Mechstiff* module of *ProDy* based on the theory introduced by Eyal and Bahar [46]. We generate an *N × N* matrix for the effective stiffness (or effective resistance or force constant, < κ*_ij_* >) for each residue pair (*i*, *j*) in response to uniaxial tension using

(4) $$\langle \kappa_{ij}\rangle =\frac{\sum_{\left(k\right)}d_{ij}^{k}\gamma \lambda_{k}}{\sum_{\left(k\right)}d_{ij}^{k}}$$

where $d_{ij}^{\left(k\right)}$ is the deformation along the kth mode in response to the tension

(5) $$d_{ij}^{\left(k\right)}={\left(k_{B}T/{\gamma \lambda }_{k}\right)}^{\frac{1}{2}}\;\;cos\alpha_{ij}{}^{\left(k\right)}|u_{i}^{\left(k\right)}-u_{j}^{\left(k\right)}|$$

Here, $\alpha_{ij}{}^{\left(k\right)}$ is the angle between the direction of the external force and that of the change $\Delta R_{ij}^{\left(k\right)}$ in the inter-residue distance induced by mode k.

*Essential site-scanning analysis* (*ESSA*) provides a measure of the change in global dynamics in response to ligand binding [47]. Ligand binding to a given residue is mimicked by crowding the neighborhood of that residue upon the inclusion of additional nodes at its side chain atoms’ positions. The *ESSA* score for each residue corresponds to the Z-score of the percent shift in the eigenvalues of the softest 10 modes due to this crowding. Higher *ESSA* scores suggest essential sites that alter the global dynamics.

*Z-scores*. For each of the above six features, we computed six values: the minimum, maximum, mean, minimum *Z-score*, maximum *Z-score*, and <*Z-score*>, considering all residues in the mAA-del segment. The *Z-score* for each feature *f* of each mAA-del residue i is computed using

(6) $$Z\text{-}score_{i}^{f}=\frac{f_{i}-\mu_{f}}{\sigma_{f}}$$

where $\mu_{f}$ and $\sigma_{f}$ denote the mean and standard deviation of f over all residues in the wt protein.

*Hinge sites*. Hinge sites in a given mode are residues distinguished by their minimal motions, if any, in that particular mode. They serve as anchors between substructures that concertedly move around them, and as such, they play a critical mechanical role. In the GNM analysis, they are readily identified by plotting the eigenvectors as a function of residue index and examining the zero-crossover points. Here, we focused on the global hinges, i.e., considering 2% of GNM modes at the lowest frequency end of the mode spectrum. We used the *calcHinges* function of *ProDy* with the default parameters and protocol to compute the hinge sites corresponding to these global modes.

### 4.3. Construction of PU Learning-Based Classifiers

We adhered to the PU learning-based classifier introduced by Elkan and Noto [68]. In previous work (*Profound*), we considered separate subsets to train classifiers for loop or non-loop regions. Here, we constructed a more robust RF classifier using the merged dataset composed of all mAA-dels (loops and non-loops) to train the classifier. Previous work [28] considered 39 (41 for loop mAA-dels) features composed of fold attributes, environment-specific properties, and deletion site-specific properties to train the classifier; here, we additionally included intrinsic dynamics-dependent attributes. We used the scikit-learn [69] implementation of the RF method, and our API *ProDy* [40,41] to evaluate dynamics-based features for all members of our dataset. We constructed nine PU learning-based classifiers (the performance of which on 10-fold stratified cross-validations is illustrated in Figure 4) corresponding to mAA-dels in the loop, non-loop, and combined datasets, each constructed with only intrinsic dynamics-based attributes, attributes used in *Profound*, and a combination of all attributes. The feature vectors corresponding to the positive and unlabeled mAA-dels in each dataset were considered to construct each PU learning-based classifier. The PU classification framework was constructed with the help of two random forest classifiers (RFCs). The first RFC consults the distribution of the different features in the positive mAA-dels to identify the likelihood of each unlabeled mAA-dels belonging to a positive and negative class. Subsequently, along with the positive mAA-dels, the unlabeled mAA-dels weighted by these probabilities are used to train the final RFC. The detailed algorithm describing the construction of the PU learning classifiers can be found in our previous work [28].

We further used the *feature_importances_* attribute from the RandomForestClassifier class of the sklearn package [69] to compute the contribution of individual features to classification. The importance of each attribute is computed as the mean and standard deviation of the decrease in the accumulation of impurity within each tree across all trees considered in the RFCs.

## Data Availability

This paper analyzed publicly available data. Any additional information required to reanalyze the data reported in this paper is available from the corresponding authors upon request (anupam.banerjee@stonybrook.edu and bahar@laufercenter.org).

## Supplementary Materials

The supporting information can be downloaded at: https://www.mdpi.com/article/10.3390/ijms24098450/s1.
