# Supplementary material for: Structural Dynamics Predominantly Determine the Adaptability of Proteins to Amino Acid Deletions
Source: Int J Mol Sci. 2023 May 8;24(9):8450. doi: 10.3390/ijms24098450 (PMC10179678; doi:10.3390/ijms24098450)
Supplement: Supplementary file 1 [file ijms-24-08450-s001.zip › ijms-2334251-supplementary.pdf]

Supplementary Information for

Structural Dynamics Predominantly Determine the Adaptability of Proteins to Amino Acid Deletions

Anupam Banerjee [1,*] and Ivet Bahar [1,2,*]

[1] Laufer Center for Physical and Quantitative Biology, Stony Brook University, Stony Brook, NY 11794, USA
[2] Department of Biochemistry and Cell Biology, Stony Brook University, Stony Brook, NY 11794, USA
[*] Correspondence: anupam.banerjee@stonybrook.edu (A.B.); bahar@laufercenter.org (I.B.)

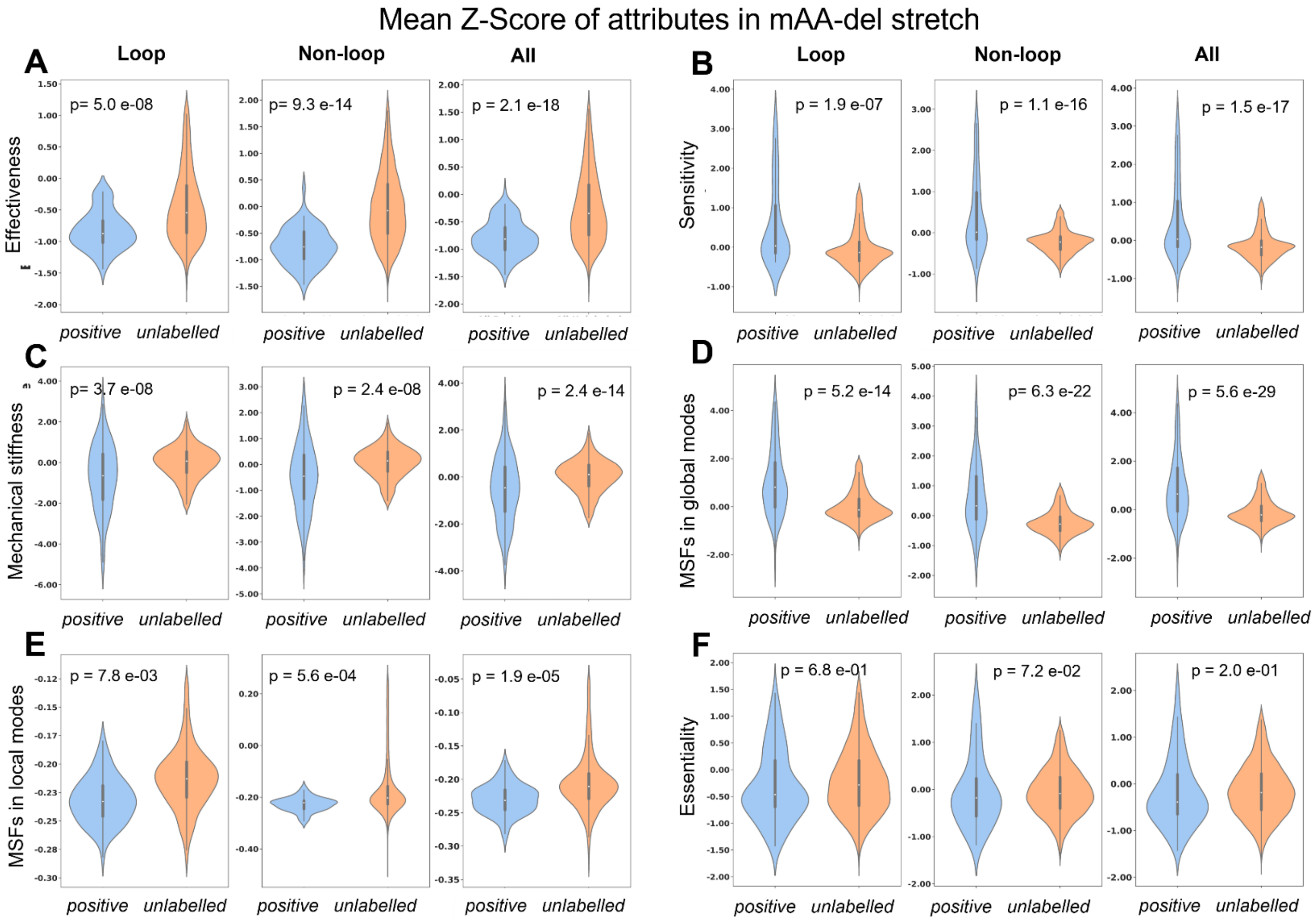

Figure S1. The <Z-score> for dynamics-based features of mAA-del residues (prior to deletion) are differentially distributed in the positive and unlabeled mAA-del subsets. The violin plots show the distribution of <Z-Scores> (averaged over the n_{AA} residues belonging to each mAA-del) for (**A**) *effectiveness,* (**B**) *sensitivity*, (**C**) *mechanical stiffness,* (**D**) *MSFs in (global modes,* (**E**) *MSFs in local modes,* and (**F**) *essentiality* (*ESSA score*) for positive and unlabeled mAA-dels in the loop, non-loop and combined dataset.

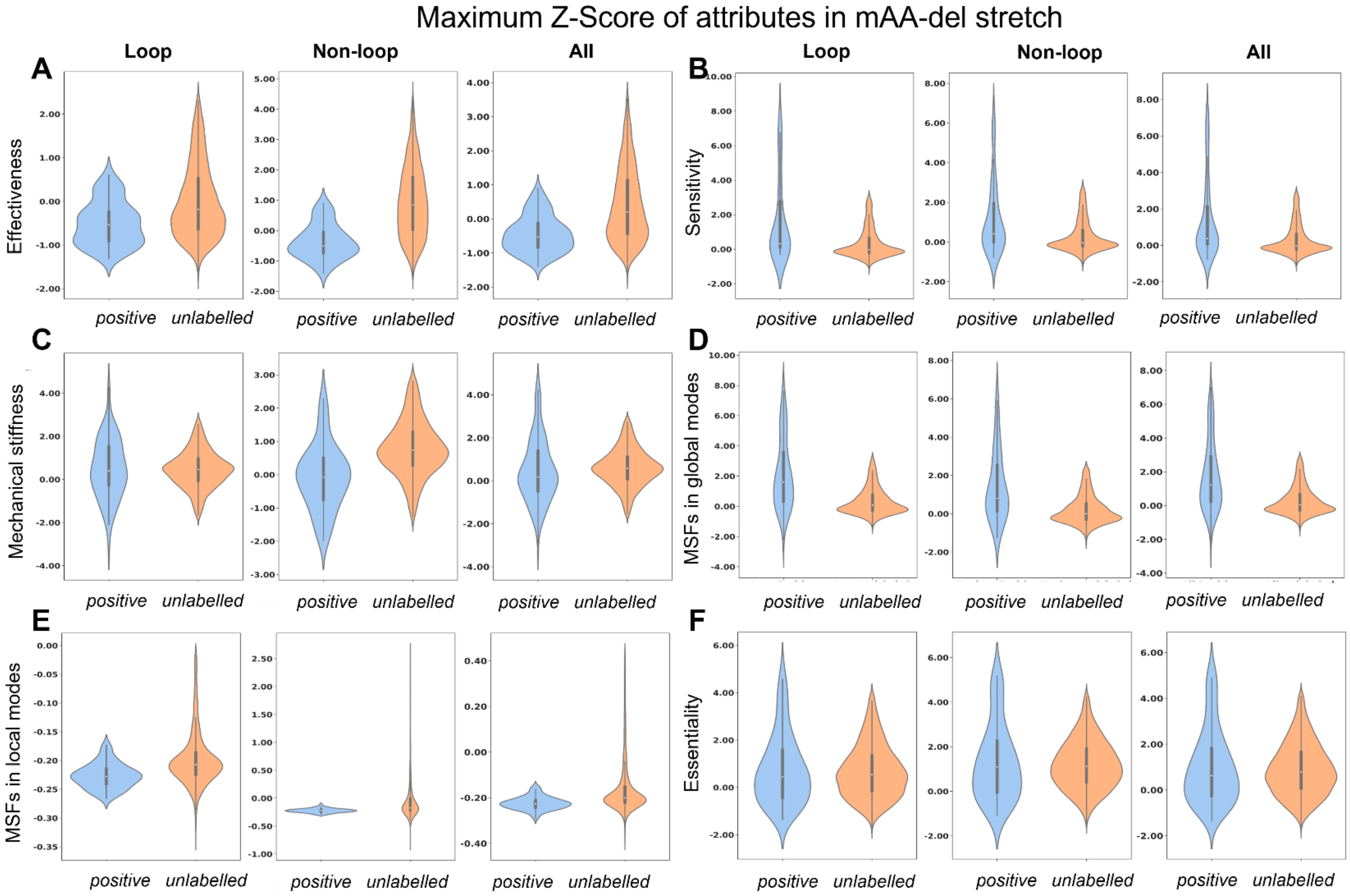

Figure S2. Differential distribution of maximum Z-Scores associated with dynamics-based features between the positive and unlabeled mAA-del subsets. The violin plots show the distribution of maximum Z-Scores (averaged over all residues in each mAA-del) for (**A**) *effectiveness,* (**B**) *sensitivity,* (**C**) *mechanical stiffness,* (**D**) *MSFs in (global modes,* (**E**) *MSFs in local modes,* and (**F**) *essentiality* (*ESSA score*) for positive and unlabeled mAA-dels in the loop, non-loop and combined dataset.

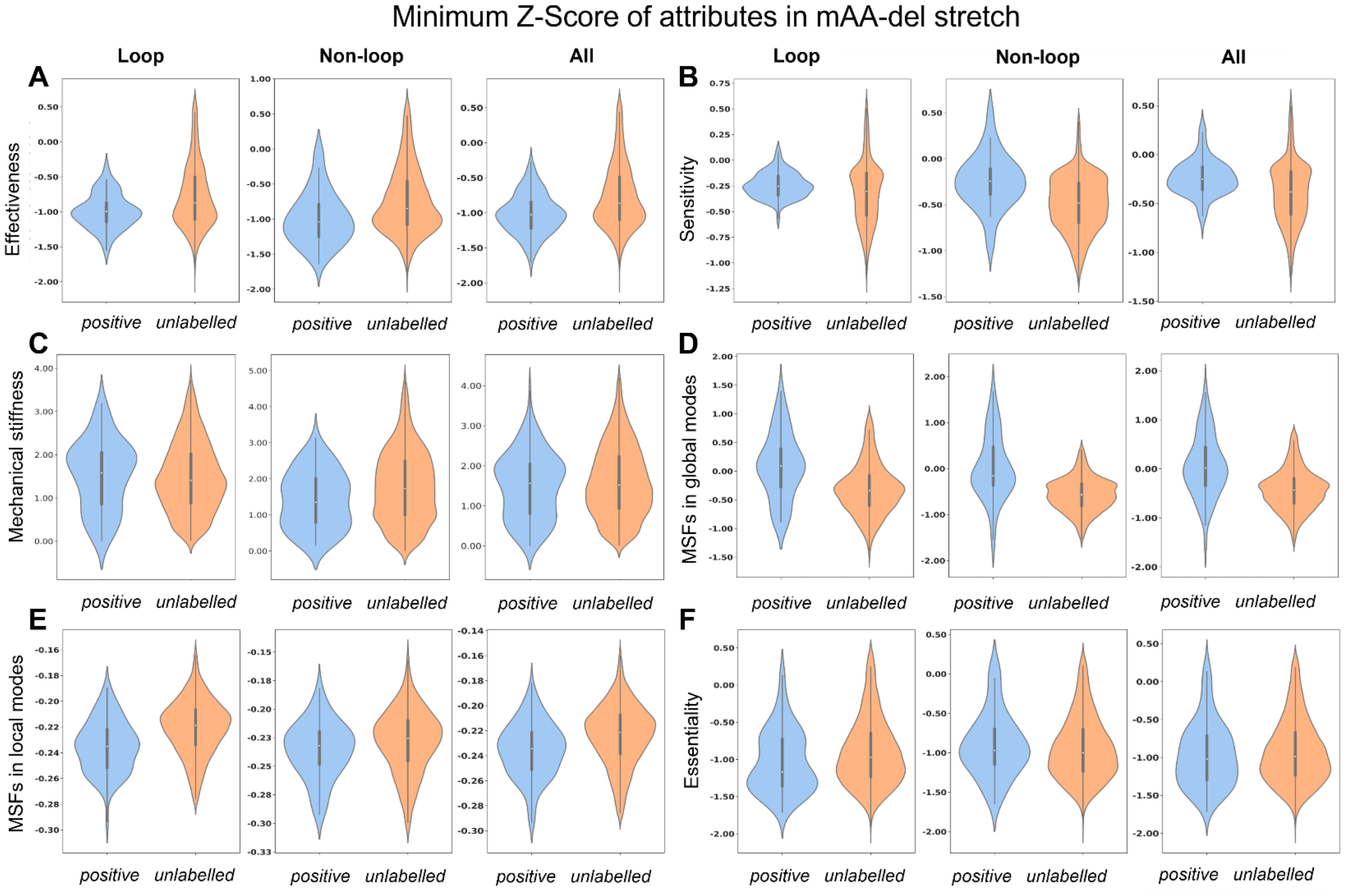

Figure S3. Differential distribution of minimum Z-Scores associated with dynamics-based features between the positive and unlabeled mAA-del subsets. The violin plots show the distribution of minimum Z-Scores (averaged over all residues in each mAA-del) for (**A**) *effectiveness,* (**B**) *sensitivity,* (**C**) *mechanical stiffness,* (**D**) *MSFs in (global modes,* (**E**) *MSFs in local modes,* and (**F**) *essentiality* (*ESSA score*) for positive and unlabeled mAA-dels in the loop, non-loop and combined dataset.

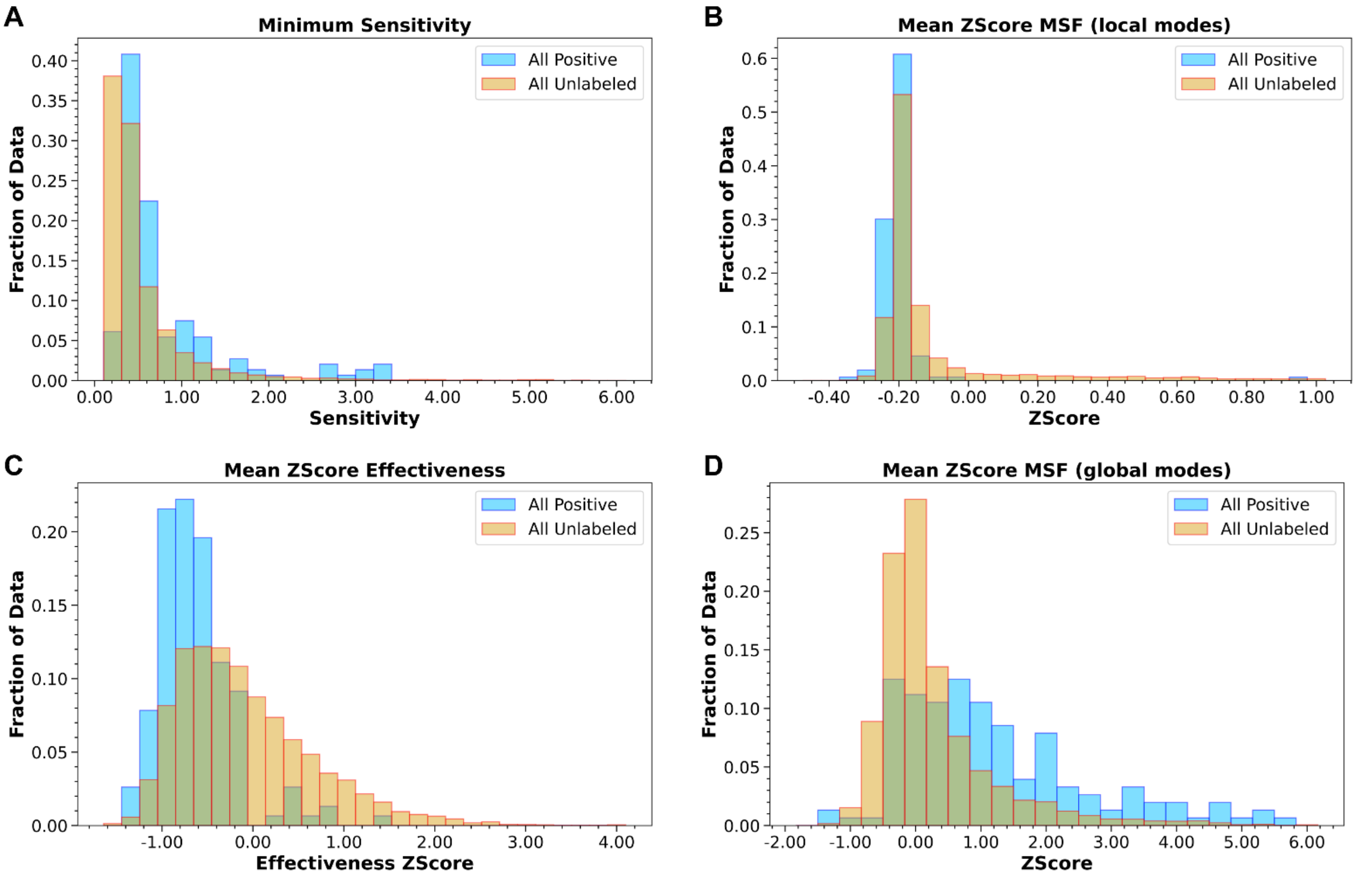

Figure S4. Distinctive distribution of dynamics-based features for positive and unlabeled mAA-dels. Histograms for the maximally contributing features (highest bar of each color from Figure 5A) from the four highest contributing types of dynamics-based features (Figure 5B). The distribution are plotted for (**A**) minimum sensitivity, (**B**) MSFs (local modes) <Z-Score>, (**C**) effectiveness < Z-Score> , and (**D**) MSFs in global modes <Z-score> as observed in the positive and unlabeled subsets of the combined set (of mAA-dels in loops and non-loops).

Table S1. Median and mean±SD of dynamics features for all positive and unlabeled mAA-dels in the loop, non-loop, and combined datasets

Features		Median						Mean±SD					
		Loop		Non-loop		Combined		Loop		Nonloop		Combined	
		P*	U	P	U	P	U	P	U	P	U	P	U
Effectiveness	Min#	0.03	0.18	0.03	0.22	0.03	0.19	0.13±0.24	0.71±2.52	0.23±0.35	0.60±1.80	0.17±0.30	0.66±2.24
	Max	0.46	0.57	0.52	1.09	0.48	0.79	0.52±0.49	1.60±6.12	0.70±0.75	2.45±6.99	0.60±0.62	1.96±6.52
	Mean	0.22	0.37	0.26	0.64	0.24	0.48	0.30±0.33	1.07±3.49	0.43±0.51	1.28±2.91	0.35±0.42	1.16±3.26
	MinZ	-0.99	-0.84	-1.02	-0.81	-1.00	-0.83	-0.96 ±0.32	-0.68±0.64	-0.93±0.49	-0.64±0.66	-0.95±0.41	-0.66±0.65
	MaxZ	-0.53	-0.16	-0.42	0.87	-0.51	0.23	-0.50 ±0.50	0.09±0.98	-0.28±0.66	1.03±1.33	-0.41±0.58	0.49±1.23
	< Z >	-0.84	-0.51	-0.73	-0.05	-0.79	-0.33	-0.77±0.37	-0.34±0.73	-0.65±0.51	0.05±0.75	-0.72±0.44	-0.17±0.76
Sensitivity	Min	0.45	0.33	0.44	0.19	0.45	0.26	1.09±1.93	0.96±7.89	0.90±1.42	0.36±1.59	1.01±1.73	0.70±6.05
	Max	1.25	0.78	1.45	0.79	1.32	0.79	9.67 ±27.03	7.34±109.64	5.33±15.49	1.92±11.49	7.80±22.88	5.00±83.07
	Mean	0.82	0.55	0.92	0.43	0.85	0.49	3.79±7.24	2.65±25.96	2.33±5.10	0.85±3.76	3.16±6.44	1.87±19.75
	MinZ	-0.24	-0.26	-0.21	-0.47	-0.23	-0.35	0.08±1.11	-0.14±0.72	-0.03±0.85	-0.44±0.48	0.04±1.01	-0.27±0.64
	MaxZ	0.51	0.08	0.46	0.02	0.48	0.06	2.04±2.94	0.80±1.80	1.79±2.74	0.65±1.52	1.93±2.86	0.73±1.68
	< Z >	0.05	-0.10	0.04	-0.19	0.04	-0.14	0.83±1.53	0.24±1.03	0.64±1.24	-0.06±0.67	0.75±1.41	0.11±0.90
Mechanical stiffness	Min	1.64	1.42	1.41	1.73	1.58	1.53	1.63±0.92	1.51±0.84	1.45±0.82	1.80±1.00	1.55±0.89	1.64±0.92
	Max	2.03	1.52	1.61	1.90	1.85	1.65	1.88±0.94	1.61±0.87	1.64±0.89	1.96±1.06	1.78±0.93	1.76±0.97
	Mean	1.95	1.47	1.53	1.82	1.73	1.59	1.75±0.92	1.56±0.85	1.55±0.85	1.88±1.03	1.66±0.90	1.70±0.95
	MinZ	-1.49	-0.38	-0.99	-0.51	-1.31	-0.43	-1.79 ±2.11	-0.65±1.33	-1.46±1.78	-0.70±1.09	-1.64±1.98	-0.67±1.23
	MaxZ	0.33	0.48	0.10	0.75	0.15	0.60	0.33±1.59	0.52±1.17	0.42±1.74	0.86±1.04	0.37±1.66	0.67±1.13
	< Z >	-0.65	0.04	-0.46	0.11	-0.49	0.08	-0.71±1.60	-0.10±1.02	-0.50±1.42	0.02±0.73	-0.62±1.53	-0.05±0.91
MSF (Global	Min	0.01	0.01	0.02	0.01	0.02	0.01	0.04±0.07	0.02±0.05	0.03±0.02	0.01±0.03	0.03±0.05	0.02±0.04
	Max	0.04	0.02	0.05	0.02	0.04	0.02	0.10±0.16	0.04±0.09	0.06±0.06	0.03±0.05	0.08±0.13	0.04±0.08
	Mean	0.03	0.02	0.03	0.01	0.03	0.01	0.07±0.10	0.03±0.06	0.04±0.04	0.02±0.04	0.06±0.08	0.03±0.05
	MinZ	0.15	-0.30	-0.15	-0.54	0.07	-0.40	0.31±1.03	-0.13±0.83	0.15±0.91	-0.47±0.59	0.24±0.98	-0.28±0.76
	MaxZ	1.61	0.18	0.88	0.09	1.25	0.14	2.16±2.28	0.78±1.73	1.57±2.09	0.49±1.27	1.90±2.22	0.66±1.55
	< Z >	0.83	-0.07	0.44	-0.25	0.67	-0.17	1.17±1.52	0.26±1.11	0.81±1.35	-0.08±0.72	1.02±1.46	0.11±0.97
MSF (Local Modes)	Min	2.5E-07	9.8E-07	3.7E-07	1.8E-06	2.6E-07	1.3E-06	3.3E-06 ±9.9E-06	4.5E-05 ±4.7E-04	5.8E-06±2.1E-05	4.8E-05±3.6E-04	4.3E-06±1.6E-05	4.6E-05±4.2E-04
	Max	1.2E-05	4.7E-05	1.4E-05	3.7E-04	1.4E-05	1.1E-04	6.1E-05 ±2.4E-04	1.6E-03 ±5.0E-03	2.8E-04±8.8E-04	4.8E-03±8.7E-03	1.6E-04±6.1E-04	3.0E-03±7.0E-03
	Mean	4.2E-06	1.9E-05	4.8E-06	1.0E-04	4.2E-06	4.1E-05	2.0E-05 ±6.2E-05	4.1E-04 ±1.5E-03	9.9E-05±3.9E-04	8.6E-04±1.9E-03	5.4E-05±2.6E-04	6.1E-04±1.7E-03
	MinZ	-0.24	-0.22	-0.23	-0.23	-0.24	-0.22	-0.24±0.02	-0.21±0.17	-0.24±0.06	-0.23±0.12	-0.24±0.04	-0.22±0.15
	MaxZ	-0.23	-0.20	-0.22	-0.12	-0.22	-0.19	-0.22±0.07	0.29±1.64	-0.15±0.32	1.15±2.43	-0.19±0.22	0.66±2.06
	< Z >	-0.23	-0.21	-0.22	-0.19	-0.23	-0.20	-0.23±0.03	-0.09±0.50	-0.21±0.15	0.01±0.52	-0.22±0.10	-0.05±0.51
E	Min	-1.04	-0.94	-0.94	-0.98	-0.97	-0.96	-0.80 ±0.87	-0.79±0.68	-0.76±0.66	-0.87±0.54	-0.78±0.78	-0.82±0.63

	**Max**	0.52	0.60	1.12	1.15	0.63	0.83	1.03±1.90	0.87±1.48	1.37±1.79	1.28±1.25	1.17±1.86	1.05±1.40
	**Mean**	-0.44	-0.25	-0.16	-0.07	-0.30	-0.16	-0.05 ±1.13	-0.09±0.88	0.13±1.03	0.00±0.60	0.03±1.09	-0.05±0.77
	**MinZ**	-1.04	-0.94	-0.94	-0.98	-0.97	-0.96	-0.80 ±0.87	-0.79±0.68	-0.76±0.66	-0.87±0.54	-0.78±0.78	-0.82±0.63
	**MaxZ**	0.52	0.60	1.12	1.15	0.63	0.83	1.03±1.90	0.87±1.48	1.37±1.79	1.28±1.25	1.17±1.86	1.05±1.40
	**< Z >**	-0.44	-0.25	-0.16	-0.07	-0.30	-0.16	-0.05 ±1.13	-0.09±0.88	0.13±1.03	0.00±0.60	0.03±1.09	-0.05±0.77

*P stands for positive mAA-dels and U stands for unlabeled mAA-dels

[#]Min, Max, Mean, MinZ, MaxZ and <Z> represent minimum, maximum, mean, minimum ZScore, maximum ZScore and mean ZScore values of individual features for all residues within a given mAA-del, based on the subsets of data.

Table S2. Statistical testing (two population hypothesis testing) of all dynamics features computed for positive and unlabeled mAA-dels in the loop, non-loop, and combined datasets

Features		t-test (Equal variance)								t-test (Unequal variance)						Z-test (Equal Variance)			
		Loop		Nonloop		Combined		Loop		Nonloop		Combined		Loop		Nonloop		Combined	
		t*	p	t	p	t	p	t	p	t	p	t	p	t	p	t	p	t	p
Effectiveness	Min	-2.13	3.3E-02	-1.69	9.1E-02	-2.70	6.9E-03	-12.45	1.2E-32	-7.02	7.3E-11	-13.92	6.4E-39	-2.13	3.3E-02	-1.69	9.1E-02	-2.70	6.9E-03
	Max	-1.64	1.0E-01	-2.02	4.3E-02	-2.58	9.8E-03	-10.08	5.2E-23	-11.38	1.4E-26	-15.14	3.9E-48	-1.64	1.0E-01	-2.02	4.3E-02	-2.58	9.8E-03
	Mean	-2.08	3.8E-02	-2.38	1.7E-02	-3.08	2.1E-03	-12.21	1.3E-31	-10.54	2.0E-20	-16.04	1.3E-49	-2.08	3.8E-02	-2.38	1.7E-02	-3.08	2.1E-03
	Min Z	-4.13	3.8E-05	-3.60	3.3E-04	-5.47	4.6E-08	-7.88	4.2E-12	-4.72	1.2E-05	-8.55	7.3E-15	-4.13	3.7E-05	-3.60	3.2E-04	-5.47	4.5E-08
	Max Z	-5.56	2.9E-08	-7.99	1.8E-15	-8.97	3.5E-19	-10.50	8.4E-18	-15.43	4.3E-25	-18.17	1.3E-42	-5.56	2.7E-08	-7.99	1.3E-15	-8.97	2.8E-19
	MeanZ	-5.46	5.0E-08	-7.48	9.3E-14	-8.77	2.1E-18	-10.26	2.8E-17	-10.80	1.3E-16	-14.77	2.5E-32	-5.46	4.7E-08	-7.48	7.3E-14	-8.77	1.7E-18
Sensitivity	Min	0.16	8.7E-01	2.73	6.4E-03	0.63	5.3E-01	0.56	5.8E-01	3.02	3.6E-03	1.97	5.0E-02	0.16	8.7E-01	2.73	6.3E-03	0.63	5.3E-01
	Max	0.20	8.4E-01	2.37	1.8E-02	0.42	6.8E-01	0.70	4.9E-01	1.77	8.2E-02	1.34	1.8E-01	0.20	8.4E-01	2.37	1.8E-02	0.42	6.8E-01
	Mean	0.41	6.8E-01	3.13	1.7E-03	0.80	4.2E-01	1.30	2.0E-01	2.32	2.3E-02	2.26	2.5E-02	0.41	6.8E-01	3.13	1.7E-03	0.80	4.2E-01
	Min Z	2.79	5.4E-03	6.83	9.7E-12	5.68	1.4E-08	1.84	7.0E-02	3.90	2.3E-04	3.70	3.0E-04	2.79	5.3E-03	6.83	8.2E-12	5.68	1.3E-08
	Max Z	6.30	3.3E-10	5.92	3.5E-09	8.58	1.2E-17	3.91	1.8E-04	3.36	1.3E-03	5.16	7.5E-07	6.30	3.0E-10	5.92	3.2E-09	8.58	9.7E-18
	MeanZ	5.22	1.9E-07	8.34	1.1E-16	8.55	1.5E-17	3.55	6.2E-04	4.58	2.2E-05	5.55	1.2E-07	5.22	1.8E-07	8.34	7.7E-17	8.55	1.3E-17
Mechanical Stiffness	Min	1.26	2.1E-01	-2.82	4.8E-03	-1.14	2.5E-01	1.14	2.6E-01	-3.39	1.2E-03	-1.19	2.4E-01	1.26	2.1E-01	-2.82	4.8E-03	-1.14	2.5E-01
	Max	2.83	4.7E-03	-2.43	1.5E-02	0.17	8.6E-01	2.60	1.1E-02	-2.88	5.4E-03	0.18	8.6E-01	2.83	4.6E-03	-2.43	1.5E-02	0.17	8.6E-01
	Mean	2.08	3.7E-02	-2.55	1.1E-02	-0.40	6.9E-01	1.92	5.8E-02	-3.03	3.4E-03	-0.42	6.7E-01	2.08	3.7E-02	-2.55	1.1E-02	-0.40	6.9E-01
	Min Z	-7.80	7.9E-15	-5.47	5.0E-08	-9.52	2.3E-21	-5.00	3.0E-06	-3.41	1.1E-03	-6.04	1.1E-08	-7.80	6.4E-15	-5.47	4.6E-08	-9.52	1.8E-21
	Max Z	-1.54	1.2E-01	-3.33	8.8E-04	-3.23	1.2E-03	-1.14	2.6E-01	-2.01	4.8E-02	-2.22	2.8E-02	-1.54	1.2E-01	-3.33	8.7E-04	-3.23	1.2E-03
	MeanZ	-5.52	3.7E-08	-5.59	2.4E-08	-7.64	2.4E-14	-3.56	5.9E-04	-2.96	4.3E-03	-4.63	7.6E-06	-5.52	3.5E-08	-5.59	2.2E-08	-7.64	2.1E-14
MSF	Min	3.09	2.0E-03	3.14	1.7E-03	4.23	2.4E-05	2.22	2.9E-02	4.39	3.9E-05	3.37	9.4E-04	3.09	2.0E-03	3.14	1.7E-03	4.23	2.3E-05
	Max	5.32	1.1E-07	4.41	1.1E-05	6.68	2.6E-11	3.21	1.8E-03	3.99	1.6E-04	4.23	4.0E-05	5.32	1.0E-07	4.41	1.0E-05	6.68	2.4E-11

	Mean	5.05	4.6E-07	4.38	1.2E-05	6.46	1.1E-10	3.16	2.2E-03	4.61	1.8E-05	4.32	2.7E-05	5.05	4.5E-07	4.38	1.2E-05	6.46	1.0E-10
	MinZ	4.78	1.8E-06	8.38	7.8E-17	8.28	1.4E-16	3.88	2.0E-04	5.52	6.0E-07	6.44	1.4E-09	4.78	1.7E-06	8.38	5.4E-17	8.28	1.2E-16
	MaxZ	7.29	3.7E-13	6.68	2.8E-11	9.71	3.8E-22	5.55	3.0E-07	4.12	1.1E-04	6.87	1.5E-10	7.29	3.2E-13	6.68	2.4E-11	9.71	2.8E-22
	MeanZ	7.55	5.2E-14	9.69	6.3E-22	11.22	5.6E-29	5.54	3.1E-07	5.31	1.4E-06	7.60	2.6E-12	7.55	4.3E-14	9.69	3.3E-22	11.22	3.3E-29
MSF (Local Modes)	Min	-0.84	4.0E-01	-0.95	3.4E-01	-1.23	2.2E-01	-5.89	4.0E-09	-6.19	7.4E-10	-8.40	5.3E-17	-0.84	4.0E-01	-0.95	3.4E-01	-1.23	2.2E-01
	Max	-2.83	4.7E-03	-4.22	2.5E-05	-4.95	7.5E-07	-18.93	9.1E-76	-24.27	9.8E-88	-29.76	1.4E-157	-2.83	4.6E-03	-4.22	2.4E-05	-4.95	7.4E-07
	Mean	-2.47	1.4E-02	-3.32	9.2E-04	-4.08	4.6E-05	-16.75	8.9E-61	-13.18	3.4E-26	-19.39	7.9E-63	-2.47	1.4E-02	-3.32	9.1E-04	-4.08	4.5E-05
	MinZ	-1.36	1.7E-01	-1.18	2.4E-01	-1.78	7.5E-02	-7.07	7.2E-12	-2.47	1.6E-02	-5.90	1.2E-08	-1.36	1.7E-01	-1.18	2.4E-01	-1.78	7.5E-02
	MaxZ	-2.92	3.5E-03	-4.36	1.4E-05	-5.12	3.1E-07	-19.67	8.6E-82	-22.61	1.5E-66	-29.02	1.6E-138	-2.92	3.5E-03	-4.36	1.3E-05	-5.12	3.1E-07
	MeanZ	-2.66	7.9E-03	-3.45	5.6E-04	-4.27	1.9E-05	-17.46	1.5E-64	-11.00	4.9E-19	-17.87	1.8E-51	-2.66	7.8E-03	-3.45	5.5E-04	-4.27	1.9E-05
ESSA	Min	-0.14	8.9E-01	1.56	1.2E-01	0.77	4.4E-01	-0.11	9.1E-01	1.29	2.0E-01	0.62	5.4E-01	-0.14	8.9E-01	1.56	1.2E-01	0.77	4.4E-01
	Max	1.00	3.2E-01	0.56	5.8E-01	1.12	2.6E-01	0.78	4.4E-01	0.39	7.0E-01	0.85	4.0E-01	1.00	3.2E-01	0.56	5.8E-01	1.12	2.6E-01
	Mean	0.41	6.8E-01	1.80	7.2E-02	1.28	2.0E-01	0.32	7.5E-01	1.06	2.9E-01	0.91	3.6E-01	0.41	6.8E-01	1.80	7.2E-02	1.28	2.0E-01
	MinZ	-0.14	8.9E-01	1.56	1.2E-01	0.77	4.4E-01	-0.11	9.1E-01	1.29	2.0E-01	0.62	5.4E-01	-0.14	8.9E-01	1.56	1.2E-01	0.77	4.4E-01
	MaxZ	1.00	3.2E-01	0.56	5.8E-01	1.12	2.6E-01	0.78	4.4E-01	0.39	7.0E-01	0.85	4.0E-01	1.00	3.2E-01	0.56	5.8E-01	1.12	2.6E-01
	MeanZ	0.41	6.8E-01	1.80	7.2E-02	1.28	2.0E-01	0.32	7.5E-01	1.06	2.9E-01	0.91	3.6E-01	0.41	6.8E-01	1.80	7.2E-02	1.28	2.0E-01

**t stands for t-statistics and p stands for p-value*

Table S3. 5-fold and 10-fold cross-validation results of the proposed PU-learning classifiers

Dataset (feature set)	5-fold CV		10-fold CV	
	**Recall (%)**	**Fall-out Rate (%)**	**Recall (%)**	**Fall-out Rate (%)**
**Loop (ProDy)**	76.24 ± 12.62	17.48 ± 1.68	78.04 ± 13.62	17.00 ± 2.01
**Loop (Profound)**	81.84 ± 9.06	14.50 ± 1.44	82.19 ± 13.03	14.19 ± 1.88
**Loop (ProDy+Profound)**	84.01 ± 8.27	16.06 ± 1.66	86.76 ± 10.96	16.08 ± 1.79
**Non-loop (ProDy)**	82.70 ± 9.72	21.62 ± 1.98	83.81 ± 13.12	21.09 ± 2.44
**Non-loop (Profound)**	84.88 ± 11.19	20.76 ± 1.87	86.60 ± 12.79	20.61 ± 2.30
**Non-loop (ProDy+Profound)**	86.76 ± 9.24	19.98 ± 1.90	88.33 ± 12.01	19.94 ± 2.24
**Combined (ProDy)**	73.92 ± 6.29	19.94 ± 1.39	78.05 ± 9.93	19.75 ± 1.54
**Combined (Profound)**	80.42 ± 7.80	15.87 ± 1.50	81.00 ± 10.03	15.40 ± 1.30
**Combined (ProDy+Profound)**	82.79 ± 6.45	18.87 ± 1.46	84.26 ± 9.19	18.26 ± 1.39

Table S4. Percentage contribution of individual feature sets to foldability classification

Feature Set	ProDy Features (%)			ProDy + Profound Features (%)		
	Loop	Nonloop	Combined	Loop	Nonloop	Combined
Effectiveness	15.63	26.23	19.67	12.00	20.09	14.98
Sensitivity	18.42	12.79	17.40	13.29	11.30	12.00
Mechanical Stiffness	11.78	10.61	12.16	8.25	6.98	7.42
MSF (Global Modes)	19.93	26.99	19.48	13.92	21.61	15.92
MSF (Local Modes)	23.53	16.27	20.95	15.75	13.92	15.54
ESSA	9.96	6.45	9.40	6.36	4.45	5.87
Hinge	0.75	0.66	0.96	0.52	0.54	0.58
Deletion site properties (Profound)	-	-	-	20.43	15.86	19.02
Environmental Compatibility (Profound)	-	-	-	7.93	3.08	6.11
Folding free energy (Profound)	-	-	-	1.55	2.16	2.56

Table S5. Percentage contribution of individual features to foldability classification

		ProDy Features (%)			ProDy + Profound Features (%)		
	Features	Loop	Nonloop	Combined	Loop	Nonloop	Combined
Effectiveness	Minimum	3.57	1.31	2.23	2.45	0.92	1.58
	Maximum	1.90	2.42	2.22	1.35	2.28	1.52
	Mean	2.14	2.04	2.17	1.60	2.09	1.60
	Minimum ZScore	1.86	1.13	1.87	1.42	0.89	1.18
	Maximum ZScore	2.85	10.24	5.55	2.53	7.34	4.53
	<Z>	3.31	9.09	5.62	2.67	6.56	4.58
Sensitivity	Minimum	3.59	5.50	4.94	2.62	4.52	3.63
	Maximum	2.66	1.08	2.35	2.11	0.91	1.40
	Mean	2.86	1.66	2.68	2.23	1.70	1.99
	Minimum ZScore	4.73	1.82	3.07	3.07	1.76	2.20
	Maximum ZScore	2.39	1.25	2.18	1.69	0.95	1.34
	<Z>	2.19	1.49	2.19	1.57	1.45	1.43
Stiffness	Minimum	1.54	1.79	1.63	1.10	1.13	0.98
	Maximum	1.70	1.68	1.64	1.30	1.09	1.00
	Mean	1.63	1.72	1.61	1.19	1.11	0.98
	Minimum ZScore	3.30	1.20	2.52	2.21	0.85	1.60
	Maximum ZScore	1.64	2.57	2.32	1.03	1.56	1.35
	<Z>	1.97	1.65	2.44	1.40	1.24	1.52
MSFs in global modes	Minimum	1.72	8.42	2.67	1.19	6.21	2.40
	Maximum	2.56	2.34	2.99	1.73	2.03	2.14
	Mean	2.20	5.69	3.47	1.64	4.67	2.87
	Minimum ZScore	3.53	5.36	3.25	2.95	4.38	3.15
	Maximum ZScore	3.89	1.65	2.60	2.40	1.31	1.84
	<Z>	6.03	3.53	4.49	4.01	3.01	3.52
	Hinge	0.75	0.66	0.96	0.52	0.54	0.58
MSFs in local modes	Minimum	2.36	3.21	3.04	1.77	3.28	2.48
	Maximum	2.39	3.26	3.05	1.73	2.53	2.13
	Mean	3.10	1.24	2.66	2.04	0.81	1.69
	Minimum ZScore	7.09	3.76	5.18	4.73	3.66	4.08
	Maximum ZScore	6.85	3.50	5.32	4.38	2.80	4.14
	<Z>	1.74	1.30	1.69	1.10	0.84	1.01
Essentiality	Minimum	1.90	1.29	1.92	1.27	0.87	1.19
	Maximum	2.11	1.22	1.83	1.32	0.84	1.17
	Mean	1.74	1.31	1.69	1.10	0.85	1.02
	Minimum ZScore	1.93	1.29	1.91	1.26	0.87	1.19

Maxi ZScore	2.12	1.20	1.83	1.31	0.83	1.17
<Z>	0.16	0.14	0.20	0.10	0.18	0.13
End to end distance	-	-	-	1.36	2.07	1.79
Salt bridge in deleted region	-	-	-	0.16	0.15	0.17
Disulphide bonds in deleted region	-	-	-	0.04	0.03	0.04
Hbond bonds in deleted region	-	-	-	0.86	1.96	1.04
Maximum phi angle in deleted region	-	-	-	1.12	0.78	1.05
Minimum phi angle in deleted region	-	-	-	1.19	0.98	1.34
Maximum psi angle in deleted region	-	-	-	1.22	0.97	1.49
Minimum psi angle in deleted region	-	-	-	1.22	0.83	1.44
Mean surface area	-	-	-	2.86	1.52	1.96
SD* surface area	-	-	-	0.34	0.26	0.33
Avg. loop propensity	-	-	-	0.98	-	-
SD loop propensity	-	-	-	0.44	-	-
ALA propensity	-	-	-	0.61	0.94	1.25
CYS propensity	-	-	-	0.11	0.10	0.12
ASP propensity	-	-	-	0.56	0.28	0.50
GLU propensity	-	-	-	0.31	0.51	0.50
PHE propensity	-	-	-	0.24	0.26	0.28
GLY propensity	-	-	-	0.98	0.40	0.73
HIS propensity	-	-	-	0.20	0.21	0.23
ILE propensity	-	-	-	0.20	0.31	0.27
LYS propensity	-	-	-	0.29	0.34	0.34
LEU propensity	-	-	-	0.81	0.41	0.57
MET propensity	-	-	-	0.13	0.14	0.14
ASN propensity	-	-	-	1.61	0.26	0.75
PRO propensity	-	-	-	0.40	0.34	0.47
GLN propensity	-	-	-	0.39	0.24	0.36
ARG propensity	-	-	-	0.26	0.30	0.32
SER propensity	-	-	-	0.57	0.27	0.43
THR propensity	-	-	-	0.34	0.27	0.34
VAL propensity	-	-	-	0.31	0.36	0.42
TRP propensity	-	-	-	0.09	0.11	0.11
TYR propensity	-	-	-	0.23	0.24	0.23
Weighted Contact Number mean	-	-	-	0.19	0.26	0.23
Weighted Contact Number SD	-	-	-	0.05	0.09	0.05
Weighted Hydrophobic Contact Number mean	-	-	-	0.42	0.40	0.47
Weighted Hydrophobic Contact Number SD	-	-	-	0.23	0.26	0.24
Salt bridge between subunits	-	-	-	0.56	0.30	0.51
Disulphide between subunits	-	-	-	0.16	0.08	0.10
Size N terminal	-	-	-	2.01	0.78	1.63

**Size C terminal**	-	-	-	4.31	0.91	2.87
**Total FoldX**	-	-	-	1.56	2.16	2.56

**SD stands for standard deviation*

Table S6. Native and mutant protein pairs in the positive mAA-del database and their existence as monomers

PDB ID#	Chain	ORGANISM	MOLECULE	MONOMERIC/ MULTIMERIC*
1M3C	A	MUS MUSCULUS	PROTO-ONCOGENE C-CRK	Biological unit of either native or mutant is monomer in the PDB
1M3B	A	MUS MUSCULUS	PROTO-ONCOGENE C-CRK	
1O1M	A	HOMO SAPIENS	HEMOGLOBIN ALPHA CHAIN	Native can exist as monomer LINK
1O1J	A	HOMO SAPIENS	HEMOGLOBIN ALPHA CHAIN	
1XV8	B	HOMO SAPIENS	ALPHA-AMYLASE	Interaction subunit of either native or mutant is monomer in Uniprot
1JXK	A	HOMO SAPIENS	ALPHA-AMYLASE, SALIVARY	
2EE5	A	HOMO SAPIENS	RHO GTPASE ACTIVATING PROTEIN 5 VARIANT	Biological unit of either native or mutant is monomer in the PDB
2EE4	A	HOMO SAPIENS	RHO GTPASE ACTIVATING PROTEIN 5 VARIANT	
2ESW	A	MUS MUSCULUS	RHO GUANINE NUCLEOTIDE EXCHANGE FACTOR 7	Biological unit of either native or mutant is monomer in the PDB
2G6F	X	RATTUS NORVEGICUS	RHO GUANINE NUCLEOTIDE EXCHANGE FACTOR 7	
2GBJ	B	HOMO SAPIENS	UBIQUITIN	Biological unit of either native or mutant is monomer in the PDB
1S1Q	D	HOMO SAPIENS	UBIQUITIN	
2GBJ	B	HOMO SAPIENS	UBIQUITIN	Biological unit of either native or mutant is monomer in the PDB
4MDK	F	HOMO SAPIENS	UBIQUITIN	
2GQG	B	HOMO SAPIENS	PROTO-ONCOGENE TYROSINE-PROTEIN KINASE ABL1	Biological unit of either native or mutant is monomer in the PDB
2HIW	B	HOMO SAPIENS	PROTO-ONCOGENE TYROSINE-PROTEIN KINASE ABL1	
2MD5	A	MUS MUSCULUS	TRANSCRIPTION FACTOR ETV6	Biological unit of either native or mutant is monomer in the PDB
2LF8	A	MUS MUSCULUS	TRANSCRIPTION FACTOR ETV6	
2UWA	A	TROPAEOLUM MAJUS	CELLULASE	Biological unit of either native or mutant is monomer in the PDB
2VH9	B	TROPAEOLUM MAJUS	CELLULASE	
2YRY	A	HOMO SAPIENS	COMPND 3 MEMBER 6	Biological unit of either native or mutant is monomer in the PDB
2D9Y	A	HOMO SAPIENS	COMPND 3 FAMILY A MEMBER 6	
2YT1	A	MUS MUSCULUS	COMPND 3 PRECURSOR PROTEIN-BINDING FAMILY B MEMBER 2	Biological unit of either native or mutant is monomer in the PDB
2YT0	A	MUS MUSCULUS	COMPND 3 PRECURSOR PROTEIN-BINDING FAMILY B MEMBER 2	
2Z24	A	ESCHERICHIA COLI	DIHYDROOROTASE	Can exist both as monomer and dimer LINK
2Z2B	A	ESCHERICHIA COLI	DIHYDROOROTASE	
2E25	A	ESCHERICHIA COLI	DIHYDROOROTASE	Can exist both as monomer and dimer LINK
2Z2B	A	ESCHERICHIA COLI	DIHYDROOROTASE	
1XGE	A	ESCHERICHIA COLI	DIHYDROOROTASE	Can exist both as monomer and dimer LINK
2Z2B	A	ESCHERICHIA COLI	DIHYDROOROTASE	

2Z28	A	ESCHERICHIA COLI	DIHYDROOROTASE	Can exist both as monomer and dimer LINK
2Z2B	A	ESCHERICHIA COLI	DIHYDROOROTASE	
2Z25	A	ESCHERICHIA COLI	DIHYDROOROTASE	Can exist both as monomer and dimer LINK
2Z2B	A	ESCHERICHIA COLI	DIHYDROOROTASE	
2Z29	A	ESCHERICHIA COLI	DIHYDROOROTASE	Can exist both as monomer and dimer LINK
2Z2B	A	ESCHERICHIA COLI	DIHYDROOROTASE	
2Z2A	A	ESCHERICHIA COLI	DIHYDROOROTASE	Can exist both as monomer and dimer LINK
2Z2B	A	ESCHERICHIA COLI	DIHYDROOROTASE	
2Z26	A	ESCHERICHIA COLI	DIHYDROOROTASE	Can exist both as monomer and dimer LINK
2Z2B	A	ESCHERICHIA COLI	DIHYDROOROTASE	
1KNB	A	HUMAN ADENOVIRUS 5	ADENOVIRUS TYPE 5 FIBER PROTEIN	Exists as trimer
4ATZ	A	UNIDENTIFIED ADENOVIRUS	FIBER PROTEIN	
2VX9	A	HALOBACTERIUM SALINARUM R1	DODECIN	Exists as dodecamer
4B2H	A	HALOBACTERIUM SALINARUM	DODECIN	
1PWK	A	RATTUS NORVEGICUS	DYNEIN LIGHT CHAIN-2	Biological unit of either native or mutant is monomer in the PDB
4D07	A	HOMO SAPIENS	DYNEIN LIGHT CHAIN 2, CYTOPLASMIC	
4G79	A	CAENORHABDITIS ELEGANS	SPINDLE ASSEMBLY ABNORMAL PROTEIN 6	Native can exist as monomer LINK
4GFA	C	CAENORHABDITIS ELEGANS	SPINDLE ASSEMBLY ABNORMAL PROTEIN 6	
2N69	A	PENTADIPLANDRA BRAZZEANA	DEFENSIN-LIKE PROTEIN	Biological unit of either native or mutant is monomer in the PDB
4HE7	A	PENTADIPLANDRA BRAZZEANA	DEFENSIN-LIKE PROTEIN	
4NUP	C	MUS MUSCULUS	N-CADHERIN EC1-2	Biological unit of either native or mutant is monomer in the PDB
2QVI	A	MUS MUSCULUS	CADHERIN-2	
3IWI	B	ESCHERICHIA COLI	BETA-LACTAMASE	Biological unit of either native or mutant is monomer in the PDB
4OLG	B	ESCHERICHIA COLI	BETA-LACTAMASE	
4F01	B	ESCHERICHIA COLI	CHAPERONE PROTEIN DNAK	Biological unit of either native or mutant is monomer in the PDB
4R5I	A	ESCHERICHIA COLI	CHAPERONE PROTEIN DNAK	
4R5L	D	ESCHERICHIA COLI	CHAPERONE PROTEIN DNAK	Native can exist as monomer LINK
4R5J	D	ESCHERICHIA COLI	CHAPERONE PROTEIN DNAK	
2X6W	A	ENTEROBACTERIA PHAGE HK620	TAIL SPIKE PROTEIN	Exists as a trimer
4XQI	A	ENTEROBACTERIA PHAGE HK620	TAIL SPIKE PROTEIN	
1IP2	A	HOMO SAPIENS	LYSOZYME C	Biological unit of either native or mutant is monomer in the PDB
1DI4	A	HOMO SAPIENS	LYSOZYME C	
1LHL	A	HOMO SAPIENS	HUMAN LYSOZYME	Biological unit of either native or mutant is monomer in the PDB
1DI4	A	HOMO SAPIENS	LYSOZYME C	
1FRS	B	ENTEROBACTERIA PHAGE FR	BACTERIOPHAGE FR CAPSID	Exists as a trimer
1FR5	C	ENTEROBACTERIA PHAGE FR	BACTERIOPHAGE FR CAPSID	
3LI0	A	METHANOTHERMOBACTER THERMAUTOTROPHICUS	OROTIDINE 5'-PHOSPHATE DECARBOXYLASE	Native can exist as monomer LINK
1LOS	D	METHANOTHERMOBACTER	OROTIDINE MONOPHOSPHATE DECARBOXYLASE	
4FX8	B	METHANOTHERMOBACTER THERMAUTOTROPHICUS STR.	OROTIDINE 5'-PHOSPHATE DECARBOXYLASE	Native can exist as monomer LINK
1LOS	D	METHANOTHERMOBACTER	OROTIDINE MONOPHOSPHATE DECARBOXYLASE	

1LZ5	A	HOMO SAPIENS	HUMAN LYSOZYME	Biological unit of either native or mutant is monomer in the PDB
1B7S	A	HOMO SAPIENS	LYSOZYME	
1LZ6	A	HOMO SAPIENS	HUMAN LYSOZYME	Biological unit of either native or mutant is monomer in the PDB
1OUH	A	HOMO SAPIENS	LYSOZYME	
1SYG	A	STAPHYLOCOCCUS AUREUS	STAPHYLOCOCCAL NUCLEASE	Biological unit of either native or mutant is monomer in the PDB
1SND	B	STAPHYLOCOCCUS AUREUS	STAPHYLOCOCCAL NUCLEASE DIMER	
1SYE	A	STAPHYLOCOCCUS AUREUS	STAPHYLOCOCCAL NUCLEASE	Biological unit of either native or mutant is monomer in the PDB
1SND	B	STAPHYLOCOCCUS AUREUS	STAPHYLOCOCCAL NUCLEASE DIMER	
1F2Z	A	STAPHYLOCOCCUS AUREUS	STAPHYLOCOCCAL NUCLEASE	Biological unit of either native or mutant is monomer in the PDB
1SND	B	STAPHYLOCOCCUS AUREUS	STAPHYLOCOCCAL NUCLEASE DIMER	
1SYC	A	STAPHYLOCOCCUS AUREUS	STAPHYLOCOCCAL NUCLEASE	Biological unit of either native or mutant is monomer in the PDB
1SND	B	STAPHYLOCOCCUS AUREUS	STAPHYLOCOCCAL NUCLEASE DIMER	
1KDC	A	STAPHYLOCOCCUS AUREUS	STAPHYLOCOCCAL NUCLEASE	Biological unit of either native or mutant is monomer in the PDB
1SND	B	STAPHYLOCOCCUS AUREUS	STAPHYLOCOCCAL NUCLEASE DIMER	
1F2M	A	STAPHYLOCOCCUS AUREUS	STAPHYLOCOCCAL NUCLEASE	Biological unit of either native or mutant is monomer in the PDB
1SND	B	STAPHYLOCOCCUS AUREUS	STAPHYLOCOCCAL NUCLEASE DIMER	
1KDB	A	STAPHYLOCOCCUS AUREUS	STAPHYLOCOCCAL NUCLEASE	Biological unit of either native or mutant is monomer in the PDB
1SND	B	STAPHYLOCOCCUS AUREUS	STAPHYLOCOCCAL NUCLEASE DIMER	
1KAA	A	STAPHYLOCOCCUS AUREUS	STAPHYLOCOCCAL NUCLEASE	Biological unit of either native or mutant is monomer in the PDB
1SND	B	STAPHYLOCOCCUS AUREUS	STAPHYLOCOCCAL NUCLEASE DIMER	
1SNC	A	STAPHYLOCOCCUS AUREUS	THERMONUCLEASE PRECURSOR	Biological unit of either native or mutant is monomer in the PDB
1SND	B	STAPHYLOCOCCUS AUREUS	STAPHYLOCOCCAL NUCLEASE DIMER	
1KDA	A	STAPHYLOCOCCUS AUREUS	STAPHYLOCOCCAL NUCLEASE	Biological unit of either native or mutant is monomer in the PDB
1SND	B	STAPHYLOCOCCUS AUREUS	STAPHYLOCOCCAL NUCLEASE DIMER	
1KAB	A	STAPHYLOCOCCUS AUREUS	STAPHYLOCOCCAL NUCLEASE	Biological unit of either native or mutant is monomer in the PDB
1SND	B	STAPHYLOCOCCUS AUREUS	STAPHYLOCOCCAL NUCLEASE DIMER	
2UY9	A	BACILLUS SUBTILIS	OXALATE DECARBOXYLASE OXDC	Biological unit of either native or mutant is monomer in the PDB
2UYA	A	BACILLUS SUBTILIS	OXALATE DECARBOXYLASE OXDC	
3LKY	A	GRIFFITHSIA	GRIFFITHSIN	Biological unit of either native or mutant is monomer in the PDB
2GTY	A	GRIFFITHSIA	GRIFFITHSIN	
3LL0	A	GRIFFITHSIA	GRIFFITHSIN	Biological unit of either native or mutant is monomer in the PDB
3LKY	A	GRIFFITHSIA	GRIFFITHSIN	
3KRA	C	MENTHA X PIPERITA	GERANYL DIPHOSPHATE SYNTHASE SMALL SUBUNIT	Native can exist as monomer LINK
3OAC	C	MENTHA X PIPERITA	GERANYL DIPHOSPHATE SYNTHASE SMALL SUBUNIT	
2NPP	C	HOMO SAPIENS	COMPND 18 ALPHA ISOFORM	Monomer (catalytic subunit) from PP2A
3P71	C	HOMO SAPIENS	COMPND 10 ALPHA ISOFORM	
3V0G	C	CIONA INTESTINALIS	VOLTAGE-SENSOR CONTAINING PHOSPHATASE	Biological unit of either native or mutant is monomer in the PDB
3V0J	B	CIONA INTESTINALIS	VOLTAGE-SENSOR CONTAINING PHOSPHATASE	

4K03	B	DROSOPHILA MELANOGASTER	CRYPTOCHROME-1	Native can exist as monomer LINK
4JZY	B	DROSOPHILA MELANOGASTER	CRYPTOCHROME-1	
4NXT	A	HOMO SAPIENS	MITOCHONDRIAL DYNAMIC PROTEIN MID51	Biological unit of either native or mutant is monomer in the PDB
4NXX	A	HOMO SAPIENS	MITOCHONDRIAL DYNAMIC PROTEIN MID51	
4OYC	B	SALMONELLA TYPHIMURIUM	LIPOPROTEIN PRGK	Biological unit of either native or mutant is monomer in the PDB
4OYC	A	SALMONELLA TYPHIMURIUM	LIPOPROTEIN PRGK	
4QK2	A	HOMO SAPIENS	CARBONIC ANHYDRASE 2	Biological unit of either native or mutant is monomer in the PDB
4QK3	A	HOMO SAPIENS	CARBONIC ANHYDRASE 2	
2HKK	A	HOMO SAPIENS	CARBONIC ANHYDRASE 2	Biological unit of either native or mutant is monomer in the PDB
4QK3	A	HOMO SAPIENS	CARBONIC ANHYDRASE 2	
5B3Y	A	HOMO SAPIENS, ESCHERICHIA COLI K-12	COMPND 3 MALTOSE-BINDING PERIPLASMIC PROTEIN	Biological unit of either native or mutant is monomer in the PDB
5B3X	A	HOMO SAPIENS, ESCHERICHIA COLI K-12	COMPND 3 MALTOSE-BINDING PERIPLASMIC PROTEIN	
4LRM	D	HOMO SAPIENS	EPIDERMAL GROWTH FACTOR RECEPTOR	Biological unit of either native or mutant is monomer in the PDB
5CAV	A	HOMO SAPIENS	EPIDERMAL GROWTH FACTOR RECEPTOR	
5CVD	A	HOMO SAPIENS	N-TERMINAL XAA-PRO-LYS N-METHYLTRANSFERASE 1	Monomeric unit in a heteromer
5E2B	B	HOMO SAPIENS	N-TERMINAL XAA-PRO-LYS N-METHYLTRANSFERASE 1	
5E8E	H	HOMO SAPIENS	THROMBIN HEAVY CHAIN	Monomeric unit in a heteromer
3GIC	B	HOMO SAPIENS	THROMBIN HEAVY CHAIN	
5EJW	A	MUS MUSCULUS	CHROMOBOX PROTEIN HOMOLOG 7	Biological unit of either native or mutant is monomer in the PDB
4X3K	A	MUS MUSCULUS	CHROMOBOX PROTEIN HOMOLOG 7	
5GQM	A	BOMBYX MORI CYPOVIRUS 1	POLYHEDRIN	Native can exist as monomer LINK
5GQJ	A	BOMBYX MORI CYPOVIRUS 1	POLYHEDRIN	
5GQI	A	BOMBYX MORI CYPOVIRUS 1	POLYHEDRIN	Native can exist as monomer LINK
5GQN	A	BOMBYX MORI CYPOVIRUS 1	POLYHEDRIN	
5EXY	A	BOMBYX MORI CYTOPLASMIC POLYHEDROSIS VIRUS	POLYHEDRIN	Native can exist as monomer LINK
5GQN	A	BOMBYX MORI CYPOVIRUS 1	POLYHEDRIN	
1HZB	A	BACILLUS CALDOLYTICUS	COLD SHOCK PROTEIN CSPB	Native can exist as monomer LINK
5JX4	B	BACILLUS CALDOLYTICUS	COLD SHOCK PROTEIN CSPB	
5JX8	B	VACCINIA VIRUS (STRAIN WESTERN RESERVE)	URACIL-DNA GLYCOSYLASE	Native can exist as monomer LINK
4IRB	B	VACCINIA VIRUS ANKARA	URACIL-DNA GLYCOSYLASE	
5K6B	F	HUMAN RESPIRATORY SYNCYTIAL VIRUS A	FUSION GLYCOPROTEIN F0	Native can exist as monomer LINK
5K6C	F	HUMAN RESPIRATORY SYNCYTIAL VIRUS A (STRAIN A2)	FUSION GLYCOPROTEIN F0	
5KC6	B	HOMO SAPIENS	CEREBELLIN-1	Native can exist as monomer LINK
5KWR	A	RATTUS NORVEGICUS	CEREBELLIN-1	
1LZ5	A	HOMO SAPIENS	HUMAN LYSOZYME	Biological unit of either native or mutant is monomer in the PDB
5LVK	B	HOMO SAPIENS	LYSOZYME C	
1LZ6	A	HOMO SAPIENS	HUMAN LYSOZYME	Biological unit of either native or mutant is monomer in the PDB
5LVK	B	HOMO SAPIENS	LYSOZYME C	
1LMT	A	HOMO SAPIENS	HUMAN LYSOZYME	Biological unit of either native or mutant is monomer in the PDB
5LVK	B	HOMO SAPIENS	LYSOZYME C	

5LVK	B	HOMO SAPIENS	LYSOZYME C	Biological unit of either native or mutant is monomer in the PDB
1DI4	A	HOMO SAPIENS	LYSOZYME C	
5MXN	f	VIBRIO CHOLERAE	TYPE VI SECRETION PROTEIN	Exists as a octadecamer
5OJQ	G	VIBRIO CHOLERAE	VIPA	
5BOZ	C	RICINUS COMMUNIS	RICIN	Disulphide linked dimer of A and B chains
5SV3	D	RICINUS COMMUNIS	RICIN	
5THF	C	INFLUENZA A VIRUS	HEMAGGLUTININ HA1 CHAIN	Native can exist as monomer LINK
5UMN	A	INFLUENZA A VIRUS	HEMAGGLUTININ	
5VG3	C	BACILLUS SUBTILIS	OXALATE DECARBOXYLASE	Exists as a hexamer
2UYA	A	BACILLUS SUBTILIS	OXALATE DECARBOXYLASE OXDC	
1K5M	B	HUMAN RHINOVIRUS 14	CHIMERA OF HRV14 COAT PROTEIN VP2 (P1B) AND THE V3 LOOP OF HIV-1 GP120	Exists as Hetero 240-mer
5W3M	C	HUMAN RHINOVIRUS 14	VIRAL PROTEIN 2	
5W57	A	PARACOCCUS DENITRIFICANS (STRAIN PD 1222)	PERIPLASMIC SOLUTE BINDING PROTEIN	Biological unit of either native or mutant is monomer in the PDB
5KZJ	A	PARACOCCUS DENITRIFICANS (STRAIN PD 1222)	PERIPLASMIC SOLUTE BINDING PROTEIN	
4RCA	A	HOMO SAPIENS	RECEPTOR-TYPE TYROSINE-PROTEIN PHOSPHATASE DELTA	Biological unit of either native or mutant is monomer in the PDB
2YD7	A	HOMO SAPIENS	PTPRD PROTEIN	
3LL0	A	GRIFFITHSIA	GRIFFITHSIN	Biological unit of either native or mutant is monomer in the PDB
2GTY	A	GRIFFITHSIA	GRIFFITHSIN	
3V0E	A	CIONA INTESTINALIS	VOLTAGE-SENSOR CONTAINING PHOSPHATASE	Biological unit of either native or mutant is monomer in the PDB
3V0J	B	CIONA INTESTINALIS	VOLTAGE-SENSOR CONTAINING PHOSPHATASE	
4GNQ	A	RATTUS NORVEGICUS	PHOSPHOENOLPYRUVATE CARBOXYKINASE, CYTOSOLIC [GTP]	Biological unit of either native or mutant is monomer in the PDB
4GMM	A	RATTUS NORVEGICUS	PHOSPHOENOLPYRUVATE CARBOXYKINASE, CYTOSOLIC [GTP]	
1E21	A	HOMO SAPIENS	RIBONUCLEASE 1	Biological unit of either native or mutant is monomer in the PDB
4KXH	D	HOMO SAPIENS	RIBONUCLEASE PANCREATIC	
5LWF	B	BACILLUS LICHENIFORMIS	BETA-LACTAMASE	Biological unit of either native or mutant is monomer in the PDB
4BLM	A	BACILLUS LICHENIFORMIS	BETA-LACTAMASE	
1D1M	B	ENTEROBACTERIA PHAGE LAMBDA	LAMBDA CRO REPRESSOR	Native can exist as monomer LINK
1D1L	A	ENTEROBACTERIA PHAGE LAMBDA	LAMBDA CRO REPRESSOR	
2DFF	A	THERMOCOCCUS KODAKARENSIS	RIBONUCLEASE HII	Biological unit of either native or mutant is monomer in the PDB
2DFE	A	THERMOCOCCUS KODAKARENSIS	RIBONUCLEASE HII	
2DFH	A	THERMOCOCCUS KODAKARENSIS	RIBONUCLEASE HII	Biological unit of either native or mutant is monomer in the PDB
2DFF	A	THERMOCOCCUS KODAKARENSIS	RIBONUCLEASE HII	
2DFH	A	THERMOCOCCUS KODAKARENSIS	RIBONUCLEASE HII	Biological unit of either native or mutant is monomer in the PDB
2DFE	A	THERMOCOCCUS KODAKARENSIS	RIBONUCLEASE HII	
2DMF	A	HOMO SAPIENS	RING FINGER PROTEIN 25	Biological unit of either native or mutant is monomer in the PDB
2DAY	A	HOMO SAPIENS	RING FINGER PROTEIN 25	
2GBK	D	HOMO SAPIENS	UBIQUITIN	Biological unit of either native or mutant is monomer in the PDB
3EEC	A	HOMO SAPIENS	UBIQUITIN	
2KJK	A	LISTERIA INNOCUA	LIN2157 PROTEIN	Biological unit of either native or mutant is monomer in the PDB

3I1E	B	LISTERIA INNOCUA	LIN2157 PROTEIN	
3Q29	C	ESCHERICHIA COLI, HOMO SAPIENS	CHIMERIC PROTEIN	Biological unit of either native or mutant is monomer in the PDB
3Q26	A	ESCHERICHIA COLI, HOMO SAPIENS	CHIMERIC PROTEIN	
2KY9	A	BACILLUS SUBTILIS	UNCHARACTERIZED PROTEIN YDHK	Biological unit of either native or mutant is monomer in the PDB
4FIB	C	BACILLUS SUBTILIS SUBSP. SUBTILIS	UNCHARACTERIZED PROTEIN YDHK	
4O4F	A	ENTAMOEBA HISTOLYTICA	INOSITOL HEXAKISPHOSPHATE KINASE	Biological unit of either native or mutant is monomer in the PDB
4O4B	B	ENTAMOEBA HISTOLYTICA	INOSITOL HEXAKISPHOSPHATE KINASE	
104L	B	ENTEROBACTERIA PHAGE T4	T4 LYSOZYME	Biological unit of either native or mutant is monomer in the PDB
1L68	A	ENTEROBACTERIA PHAGE T4	LYSOZYME	
104L	B	ENTEROBACTERIA PHAGE T4	T4 LYSOZYME	Biological unit of either native or mutant is monomer in the PDB
171L	A	ENTEROBACTERIA PHAGE T4	T4 LYSOZYME	
3IFK	A	RATTUS NORVEGICUS	CALMODULIN	Biological unit of either native or mutant is monomer in the PDB
1AHR	A	GALLUS GALLUS	CALMODULIN	
1C7P	A	HOMO SAPIENS	LYSOZYME	Biological unit of either native or mutant is monomer in the PDB
1DI4	A	HOMO SAPIENS	LYSOZYME C	
1FRS	A	ENTEROBACTERIA PHAGE FR	BACTERIOPHAGE FR CAPSID	Exist as trimer
1FR5	C	ENTEROBACTERIA PHAGE FR	BACTERIOPHAGE FR CAPSID	
1IFG	A	ESCHERICHIA COLI	ECOTIN	Biological unit of either native or mutant is monomer in the PDB
1AZZ	C	ESCHERICHIA COLI	ECOTIN	
1MB8	A	HOMO SAPIENS	PLECTIN	Biological unit of either native or mutant is monomer in the PDB
4Q59	A	HOMO SAPIENS	PLECTIN	
1SRA	A	HOMO SAPIENS	SPARC	Biological unit of either native or mutant is monomer in the PDB
1NUB	B	HOMO SAPIENS	BASEMENT MEMBRANE PROTEIN BM-40	
1RJ7	H	HOMO SAPIENS	ECTODYSPLASIN A	Exists as trimer
1RJ8	G	HOMO SAPIENS	ECTODYSPLASIN-A ISOFORM EDA-A2	
1S16	A	ESCHERICHIA COLI	TOPOISOMERASE IV SUBUNIT B	Biological unit of either native or mutant is monomer in the PDB
1S14	B	ESCHERICHIA COLI	TOPOISOMERASE IV SUBUNIT B	
1STA	A	STAPHYLOCOCCUS AUREUS	STAPHYLOCOCCAL NUCLEASE	Biological unit of either native or mutant is monomer in the PDB
2F3W	A	STAPHYLOCOCCUS AUREUS	THERMONUCLEASE	
209L	A	ENTEROBACTERIA PHAGE T4	T4 LYSOZYME	Biological unit of either native or mutant is monomer in the PDB
210L	A	ENTEROBACTERIA PHAGE T4	T4 LYSOZYME	
2CME	B	HUMAN SARS CORONAVIRUS	HYPOTHETICAL PROTEIN 5	Exist as dimer
2CME	H	HUMAN SARS CORONAVIRUS	HYPOTHETICAL PROTEIN 5	
1M7V	A	BACILLUS SUBTILIS	NITRIC OXIDE SYNTHASE	Native can exist as monomer LINK
2FC1	A	BACILLUS SUBTILIS	NITRIC OXIDE SYNTHASE	
3AFI	E	BRADYRHIZOBIUM JAPONICUM	HALOALKANE DEHALOGENASE	Interaction subunit of either native or mutant is monomer in Uniprot
3A2L	B	BRADYRHIZOBIUM JAPONICUM	HALOALKANE DEHALOGENASE	
3AAK	A	HOMO SAPIENS	PROGRAMMED CELL DEATH PROTEIN 6	Native can exist as monomer LINK
3AAJ	A	HOMO SAPIENS	PROGRAMMED CELL DEATH PROTEIN 6	
2ZRS	C	HOMO SAPIENS	PROGRAMMED CELL DEATH PROTEIN 6	Native can exist as monomer LINK
3AAJ	A	HOMO SAPIENS	PROGRAMMED CELL DEATH PROTEIN 6	
3QDO	A	RATTUS NORVEGICUS	POTASSIUM CHANNEL 3 CHIMERA	Exists as dimer

3QE1	A	RATTUS NORVEGICUS	POTASSIUM CHANNEL 3 CHIMERA	
4E89	A	XENOTROPIC MULV-RELATED VIRUS	RNASE H	Biological unit of either native or mutant is monomer in the PDB
3V1Q	A	XENOTROPIC MULV-RELATED VIRUS VP35	REVERSE TRANSCRIPTASE/RIBONUCLEASE H P80	
4AMJ	B	MELEAGRIS GALLOPAVO	BETA-1 ADRENERGIC RECEPTOR	Biological unit of either native or mutant is monomer in the PDB
2YCX	A	MELEAGRIS GALLOPAVO	BETA-1 ADRENERGIC RECEPTOR	
2X5X	A	PAUCIMONAS LEMOIGNEI	PHB DEPOLYMERASE PHAZ7	Biological unit of either native or mutant is monomer in the PDB
4BVL	D	PAUCIMONAS LEMOIGNEI	PHB DEPOLYMERASE PHAZ7	
4LZB	I	VACCINIA VIRUS	URACIL-DNA GLYCOSYLASE	Exists as dimer
4IRB	B	VACCINIA VIRUS ANKARA	URACIL-DNA GLYCOSYLASE	
4GU5	A	DROSOPHILA MELANOGASTER	CRYPTOCHROME-1	Biological unit of either native or mutant is monomer in the PDB
4JZY	B	DROSOPHILA MELANOGASTER	CRYPTOCHROME-1	
3SJZ	A	SULFOLOBUS SOLFATARICUS P2	TRANSLATION INITIATION FACTOR 2 SUBUNIT GAMMA	Biological unit of either native or mutant is monomer in the PDB
4M53	A	SULFOLOBUS SOLFATARICUS	TRANSLATION INITIATION FACTOR 2 SUBUNIT GAMMA	
3RBB	A	HIV-1 M	PROTEIN NEF	Interaction subunit of either native or mutant is monomer in Uniprot
4ORZ	B	HIV-1 M	PROTEIN NEF	
1SH6	A	MUS MUSCULUS	PLECTIN 1	Biological unit of either native or mutant is monomer in the PDB
4Q57	B	MUS MUSCULUS	PLECTIN	
5L6T	B	HOMO SAPIENS	CARBONIC ANHYDRASE 2	Biological unit of either native or mutant is monomer in the PDB
4QK3	A	HOMO SAPIENS	CARBONIC ANHYDRASE 2	
5APY	C	SACCHAROMYCES CEREVISIAE	GENERAL CONTROL PROTEIN GCN4	Native can exist as monomer LINK
5APW	B	SACCHAROMYCES CEREVISIAE	GENERAL CONTROL PROTEIN GCN4	
5AZ8	A	ESCHERICHIA COLI (STRAIN K12)	RECEPTOR SUBUNIT TOM20 HOMOLOG	Biological unit of either native or mutant is monomer in the PDB
5AZ6	B	ESCHERICHIA COLI (STRAIN K12)	RECEPTOR SUBUNIT TOM20 HOMOLOG	
4WMC	G	KLEBSIELLA PNEUMONIAE	BETA-LACTAMASE	6PK0 Crystal Structure of OXA-48 with Hydrolyzed Imipenem is a monomer
5FDH	A	SERRATIA MARCESCENS	BETA-LACTAMASE	
3AJP	A	HOMO SAPIENS	FERRITIN HEAVY CHAIN	24-mer
5GN8	A	HOMO SAPIENS	FERRITIN HEAVY CHAIN	
3AJQ	A	HOMO SAPIENS	FERRITIN HEAVY CHAIN	24-mer
5GN8	A	HOMO SAPIENS	FERRITIN HEAVY CHAIN	
3AJP	A	HOMO SAPIENS	FERRITIN HEAVY CHAIN	24-mer
5GN8	B	HOMO SAPIENS	FERRITIN HEAVY CHAIN	
4XHS	A	HOMO SAPIENS	MALTOSE-BINDING PERIPLASMIC PROTEIN,NACHT, LRR AND PYD DOMAINS-CONTAINING PROTEIN 12	Biological unit of either native or mutant is monomer in the PDB
5H7N	B	HOMO SAPIENS	NLRP12-PYD WITH MBP TAG	
1X23	D	HOMO SAPIENS	UBIQUITIN-CONJUGATING ENZYME E2 D3	Biological unit of either native or mutant is monomer in the PDB
5IFR	A	HOMO SAPIENS	UBIQUITIN-CONJUGATING ENZYME E2 D3	
1HKX	E	MUS MUSCULUS	II ALPHA CHAIN	Native can exist as monomer LINK
5IG3	A	HOMO SAPIENS	ALPHA	
5IG6	A	HOMO SAPIENS	BROMODOMAIN-CONTAINING PROTEIN 2	Biological unit of either native or mutant is monomer in the PDB
2DVV	A	HOMO SAPIENS	BROMODOMAIN-CONTAINING PROTEIN 2	
5JBH	7	PYROCOCCUS ABYSSI GE5	AIF2-GAMMA	Biological unit of either native or mutant is monomer in the PDB

3SJZ	A	SULFOLOBUS SOLFATARICUS P2	TRANSLATION INITIATION FACTOR 2 SUBUNIT GAMMA	
5JBH	7	PYROCOCCUS ABYSSI GE5	AIF2-GAMMA	Biological unit of either native or mutant is monomer in the PDB
3PEN	A	SULFOLOBUS SOLFATARICUS	TRANSLATION INITIATION FACTOR 2 SUBUNIT GAMMA	
5JVG	M	DEINOCOCCUS RADIODURANS R1	50S RIBOSOMAL PROTEIN L19	Exists as 29-mer
5DM6	M	DEINOCOCCUS RADIODURANS	50S RIBOSOMAL PROTEIN L19	
5JVS	A	DROSOPHILA MELANOGASTER	ASSOCIATED PROTEIN RP/EB FAMILY MEMBER 1	Native can exist as monomer LINK
5JVR	F	DROSOPHILA MELANOGASTER	HUMAN EB1	
5JVU	B	DROSOPHILA MELANOGASTER, HOMO SAPIENS	ASSOCIATED PROTEIN RP/EB FAMILY MEMBER 1	Native can exist as monomer LINK
5JVR	C	MUS MUSCULUS, DROSOPHILA MELANOGASTER, HOMO	HUMAN EB1	
5KDO	A	RATTUS NORVEGICUS	GUANINE NUCLEOTIDE-BINDING PROTEIN G(I) SUBUNIT ALPHA-1	Biological unit of either native or mutant is monomer in the PDB
1GIA	A	RATTUS NORVEGICUS	G PROTEIN GI ALPHA 1	
2X0L	A	HOMO SAPIENS	LYSINE-SPECIFIC HISTONE DEMETHYLASE 1	Occurrence of monomeric state in PDB forming different heteromers
5LGU	A	HOMO SAPIENS	LYSINE-SPECIFIC HISTONE DEMETHYLASE 1A	
5LGN	A	HOMO SAPIENS	LYSINE-SPECIFIC HISTONE DEMETHYLASE 1A	Occurrence of monomeric state in PDB forming different heteromers
5LGU	A	HOMO SAPIENS	LYSINE-SPECIFIC HISTONE DEMETHYLASE 1A	
3MHY	B	AZOSPIRILLUM BRASILENSE	PII-LIKE PROTEIN PZ	Exist as trimer
5OVO	B	AZOSPIRILLUM BRASILENSE	NITROGEN REGULATORY PROTEIN P-II 1	
1IFS	A	RICINUS COMMUNIS	RICIN	Disulfide linked dimer of A and B chains
5SV3	D	RICINUS COMMUNIS	RICIN	
104L	A	ENTEROBACTERIA PHAGE T4	T4 LYSOZYME	Biological unit of either native or mutant is monomer in the PDB
5VNR	A	ENTEROBACTERIA PHAGE T4	ENDOLYSIN	
209L	A	ENTEROBACTERIA PHAGE T4	T4 LYSOZYME	Biological unit of either native or mutant is monomer in the PDB
5VNR	A	ENTEROBACTERIA PHAGE T4	ENDOLYSIN	
5W57	B	PARACOCCUS DENITRIFICANS (STRAIN PD 1222)	PERIPLASMIC SOLUTE BINDING PROTEIN	Biological unit of either native or mutant is monomer in the PDB
5KZJ	A	PARACOCCUS DENITRIFICANS (STRAIN PD 1222)	PERIPLASMIC SOLUTE BINDING PROTEIN	
5H7Q	A	HOMO SAPIENS	MNDA PYD DOMAIN WITH MBP TAG	Biological unit of either native or mutant is monomer in the PDB
5WQ6	D	HOMO SAPIENS	MBP TAGGED HMNDA-PYD	
5Y32	A	MUS MUSCULUS	RECEPTOR-TYPE TYROSINE-PROTEIN PHOSPHATASE DELTA	Biological unit of either native or mutant is monomer in the PDB
2YD7	A	HOMO SAPIENS	PTPRD PROTEIN	
5YI5	X	HOMO SAPIENS	FERRITIN HEAVY CHAIN	24-mer
5GN8	A	HOMO SAPIENS	FERRITIN HEAVY CHAIN	
5YI5	X	HOMO SAPIENS	FERRITIN HEAVY CHAIN	24-mer
5GN8	B	HOMO SAPIENS	FERRITIN HEAVY CHAIN	
1JI1	A	THERMOACTINOMYCES VULGARIS	ALPHA-AMYLASE I	Biological unit of either native or mutant is monomer in the PDB
5Z0U	A	THERMOACTINOMYCES VULGARIS	NEOPULLULANASE 1	
1ORC	A	ENTEROBACTERIA PHAGE LAMBDA	CRO REPRESSOR INSERTION MUTANT K56-[DGEVK]	Biological unit of either native or mutant is monomer in the PDB
6CRO	A	ENTEROBACTERIA PHAGE LAMBDA	LAMBDA CRO REPRESSOR	

** Evidence of existence as monomer of either the native (wt) or mutant protein is shown. The links point to articles describing the existence of the protein as monomeric units.*

# The odd numbered entries correspond to the wt and the even numbered entries correspond to the mutant protein.
